# *Syngap1* Disruption Induced by Recombination between Inverted loxP Sites Is Associated with Hippocampal Interneuron Dysfunction

**DOI:** 10.1523/ENEURO.0475-22.2023

**Published:** 2023-05-04

**Authors:** Abdessattar Khlaifia, Vidya Jadhav, Marc Danik, Théo Badra, Martin H. Berryer, Alexandre Dionne-Laporte, Bidisha Chattopadhyaya, Graziella Di Cristo, Jean-Claude Lacaille, Jacques L. Michaud

**Affiliations:** 1 Department of Neuroscience, Research Group on Neuronal Signaling and Circuits and Center for Interdisciplinary Research on Brain and Learning, University of Montreal, Montreal, Québec H3C 3J7, Canada; 2 University Hospital Center (CHU) Sainte-Justine Research Center, Montréal, Québec H3T1C5, Canada

**Keywords:** SYNGAP1, hippocampus, fast-spiking interneurons, synaptic deficits, excitatory inputs

## Abstract

*SYNGAP1* haploinsufficiency in humans causes intellectual disability (ID). SYNGAP1 is highly expressed in cortical excitatory neurons and, reducing its expression in mice accelerates the maturation of excitatory synapses during sensitive developmental periods, restricts the critical period window for plasticity, and impairs cognition. However, its specific role in interneurons remains largely undetermined. In this study, we investigated the effects of conditional *Syngap1* disruption in medial ganglionic eminence (MGE)-derived interneurons on hippocampal interneuron firing properties and excitatory synaptic inputs, as well as on pyramidal cell synaptic inhibition and synaptic integration. We show that conditional *Syngap1* disruption in MGE-derived interneurons results in cell-specific impairment of firing properties of hippocampal Nkx2.1 fast-spiking interneurons, with enhancement of their AMPA receptor (AMPAR)-mediated excitatory synaptic inputs but compromised short-term plasticity. In contrast, regular-spiking Nkx2.1 interneurons are largely unaffected. These changes are associated with impaired pyramidal cell synaptic inhibition and enhanced summation of excitatory responses. Unexpectedly, we found that the *Syngap1^flox^* allele used in this study contains inverted loxP sites and that its targeted recombination in MGE-derived interneurons induces some cell loss during embryonic development and the reversible inversion of the sequence flanked by the loxP sites in postmitotic cells. Together, these results suggest that *Syngap1* plays a role in cell-specific regulation of hippocampal interneuron function and inhibition of pyramidal cells in mice. However, because of our finding that the *Syngap1^flox^* allele used in this study contains inverted loxP sites, it will be important to further investigate interneuron function using a different *Syngap1* conditional allele.

## Significance Statement

Previous studies have shown that the intellectual disability (ID) gene *SYNGAP1* is expressed in parvalbumin GABAergic interneurons but its role in these cells remains poorly understood. Here, we found that *Syngap1* disruption in medial ganglionic eminence (MGE)-derived cells selectively affected hippocampal Nkx2.1 fast-spiking interneurons firing properties, their excitatory inputs strength and short-term plasticity, without affecting Nkx2.1 regular-spiking interneurons. These changes were associated with reduced synaptic inhibition of pyramidal cells. Unexpectedly, we found that the *Syngap1^flox^* allele used in this study contains inverted loxP sites and that its recombination induces cell loss during development and the inversion of the *Syngap1* targeted sequence in postmitotic cells. Thus, it will be important to further study interneuron function using a different *Syngap1* conditional allele.

## Introduction

Neuronal computation relies on tight interactions between excitatory and inhibitory neurons. Cortical GABAergic cells constitute a heterogeneous population of inhibitory interneurons and represent 10–20% of the total neuronal population. Interneurons ensure the proper functioning of cortical network circuits, by gating information flow, sculpting neuronal patterns and preventing their hyperexcitability (Tremblay et al., 2016; Pelkey et al., 2017). Inhibitory cell dysfunctions are linked to several brain pathologies, such as epilepsy, autism, intellectual disability (ID), Rett syndrome, anxiety disorders and schizophrenia (Rossignol, 2011; Chattopadhyaya and Cristo, 2012; Marín, 2012).

Numerous studies with mouse models suggest that mutations in genes encoding proteins involved in synaptic structure and function are implicated in neurodevelopmental disorders like intellectual disability (ID) and autism spectrum disorders (ASDs; Südhof, 2008; Zoghbi and Bear, 2012). Dysfunction of synaptic proteins affect the organization and function of neuronal circuits in the developing and mature brain. For example, haploinsufficiency of the *SYNGAP1* gene, encoding for such a protein, represents a common cause of ID with co-morbid ASD or/and epilepsy (Hamdan et al., 2009; Berryer et al., 2013, 2016). *SYNGAP1* codes for the synaptic GTPase-activating protein 1 (SYNGAP1) that negatively regulates Ras. In cortical excitatory neurons, SYNGAP1 is physically associated with the postsynaptic density 95 protein (PSD-95) and acts downstream of NMDA receptors (NMDARs; Kim et al., 1998; Walkup et al., 2016). SYNGAP1 limits the strengthening of excitatory synapses by restricting AMPA receptors (AMPARs) synthesis through inhibition of the Ras-ERK and mTOR signaling pathways (Wang et al., 2013; Araki et al., 2015) and by limiting the binding of auxiliary proteins (TARPs, LRRTM2, and neuroligin-2) to the PDZ domains of PSD-95 (Walkup et al., 2016). These actions ensure proper refinement and maturation of synaptic connections. Indeed, in *Syngap1*^+/−^ mice, excitatory neurons show increased AMPAR density, larger spine heads and premature maturation of excitatory synapses (Clement et al., 2012, 2013; Aceti et al., 2015; Jeyabalan and Clement, 2016). These changes lead to altered excitatory/inhibitory (E/I) balance and abnormal critical periods of plasticity in somatosensory cortex (Clement et al., 2012, 2013). Thus, *Syngap1* haploinsufficiency in excitatory neurons is believed to underlie the synaptic and cognitive abnormalities observed in patients and mouse models of neurodevelopmental disorders.

However, recent studies found that SYNGAP1 is also expressed in GABAergic interneurons, particularly in fast-spiking parvalbumin-expressing (PV) interneurons (Berryer et al., 2016; Su et al., 2019), which are important for brain rhythms generation, working memory and attention control (Hu et al., 2014). Cortical interneurons can be classified into two groups based on their developmental origin from the medial ganglionic eminence (MGE) or caudal ganglionic eminence (CGE; Kepecs and Fishell, 2014). *Syngap1* haploinsufficiency in MGE-derived interneurons has been reported to impair inhibitory synaptic transmission and cognition (Berryer et al., 2016), suggesting that SYNGAP1 in inhibitory cells ensures the proper formation of PV cell synaptic fields in a cell autonomous fashion (Berryer et al., 2016). In addition, the expression of the GluA2 subunit of AMPARs is upregulated in PV interneurons (PV-INs) in some brain regions like CA3 hippocampus and somatosensory barrel cortex of *Syngap1*^+/−^ mice (Sullivan et al., 2020). Although, these lines of evidence point to an important role of SYNGAP1 in interneuron development and function, its specific role in distinct types of inhibitory cells remains largely unknown.

Here, we used whole-cell patch-clamp recordings in slices to examine the role of SYNGAP1 in hippocampal CA1 MGE-derived Nkx2.1-expressing cells (Nkx2.1^+^), more specifically fast spiking [FS; likely corresponding to parvalbumin (PV) expressing interneurons] and regular spiking (RS) interneurons [likely corresponding to somatostatin (SOM) expressing interneurons; Tricoire et al., 2011; Pelkey et al., 2017]. We report that *Syngap1* disruption in these cells selectively impaired firing properties of Nkx2.1^+^ FS cells, enhanced their AMPAR-mediated excitatory synaptic inputs, and compromised the short-term dynamics of their excitatory synapses. Interestingly, Nkx2.1^+^ RS interneurons were largely unaffected. These changes in inhibitory interneurons were associated with a reduction of synaptic inhibition, as well as facilitation of excitatory postsynaptic response summation, in pyramidal cells. However, we found unexpectedly that the *Syngap1* conditional allele used in this study contains inverted loxP sites and that its recombination in MGE-derived interneurons induces some cell loss during embryonic development and the reversible inversion of the sequence flanked by the loxP sites in postmitotic cells.

## Materials and Methods

### Mice

All animal procedures and experiments were performed in accordance with the regulations of the Comité Institutionnel de Bonnes Pratiques Animales en Recherche (CIBPAR) of the Research Center of Sainte-Justine Hospital and the Animal Care Committee of Université de Montréal (CDEA). Mice were housed (two to five per cage), maintained in a 12/12 h light/dark cycle, and given *ad libitum* access to food and water. Experiments were performed during the light phase (6 A.M. to 6 P.M.). *Syngap1^flox/flox^* (*Syngap1^f/f^*) mice were kindly provided by Drs. Irene Knuesel and Mary Muhia (University of Zurich, Switzerland), maintained on a C57BL/6 background, and genotyped as described previously (Vazquez et al., 2004; Knuesel et al., 2005). A triple transgenic line was created by breeding *Syngap1^f/+^* mice to the *Nkx2.1-Cre* promoter line (The Jackson Laboratory #008661) and with *RCE^flox/flox^* mice (*Gt(ROSA)26Sor^tm1.1(CAG-EGFP)Fsh^*/^Mjax^; The Jackson laboratory stock #32037). The *RCE^flox/flox^* mouse carries a loxP flanked STOP cassette upstream of the EGFP gene, and removal of the cassette by Cre-mediated recombination drives EGFP reporter expression. The Tg(*Nkx2.1-Cre*) transgene starts to be expressed in the MGE at embryonic day (E)10.5 (Xu et al., 2008). By generating these triple transgenic Tg(*Nkx2.1-Cre;RCE^flox/flox^;Syngap1^flox/+^* mice, we aimed to decrease *Syngap1* specifically in MGE-derived cells while labeling them concurrently. In this study, we focused mainly our attention on two genotypes resulting from the triple transgenic breeding, Tg(*Nkx2.1-Cre*)*;RCE^flox/flox^*;*Syngap1^flox/+^* (termed *Nkx2.1-Syngap1^f/+^* mice) and Tg(*Nkx2.1-Cre);RCE^flox/flox^;Syngap1^+/+^* (termed *Nkx2.1-Syngap1^+/+^* mice). In both genotypes, *Nkx2.1*-expressing cells were identified by the expression of EGFP. We also bred the *PV-Cre* mouse line (The Jackson Laboratory #008069) to *RCE^flox/flox^*;*Syngap1^flox/+^* mice to generate mice with a decrease of *Syngap1* specifically in postmitotic PV cells (Tg(*PVCre*);*RCE^flx/flx^*;*Syngap1^flox/+^* termed *Pv-Syngap1^f/+^* mice; and Tg(*PVCre*);*RCE^flx/flx^*;*Syngap1^+/+^* termed *Pv-Syngap1^+/+^*). To target recombination of the *Syngap1^flox^* allele in most excitatory neurons of the cortex and hippocampus and their proliferating progenitors, we used the *Emx1-cre* mouse line (The Jackson Laboratory #005628) to generate the following genotypes: Tg(*Emx1Cre*);*Syngap1^flox/+^*(termed *Emx1-Syngap1^f/+^*), Tg(*Emx1Cre*);*Syngap1^flox/flox^* (termed *Emx1-Syngap1^f/f^* mice), and Tg(*Emx1Cre*);*Syngap1^+/+^* (termed *Emx1-Syngap1^+/+^* mice).

### Acute slice preparation

Electrophysiological recordings were performed on six- to nine-week-old mice of either sex. Acute hippocampal slices were prepared from *Nkx2.1-Syngap1^+/+^* or *Nkx2.1-Syngap1^f/+^* mice. Animals were lightly anesthetized with isoflurane, the brain was quickly removed, and 300-μm-thick coronal slices were cut in oxygenated ice-cold sucrose-based cutting solution containing (in mm) 87 NaCl, 2.5 KCl, 25 NaHCO_3_, 0.5 CaCl_2_, 1.25 NaH_2_PO_4_, 7 MgCl_2_, 25 sucrose, 75 sucrose, 3 pyruvic acid, and 1 ascorbic acid (300 mOsm and pH 7.4). Slices were then transferred in artificial cerebrospinal fluid (ACSF) containing (in mm) 124 NaCl, 5 KCl, 1.25 NaH_2_PO_4_, 2 MgCl_2_, 2 CaCl_2_, 26 NaHCO_3_, and 10 glucose (300 mOsm and pH 7.4) and allowed to recover for at least 1 h at room temperature (RT).

### Whole-cell recordings

Individual slices were transferred to a submersion-type recording chamber mounted on an upright microscope (Nikon Eclipse, E600FN) equipped with a water immersion long-working distance objective (40×), epifluorescence and an infrared video camera. Slices were continuously perfused (2 ml/min) with ACSF at 29–30°C. Whole-cell recordings were obtained from visually identified CA1 pyramidal cells or EGFP-expressing interneurons using borosilicate pipettes (3–5 MΩ). Current-clamp recordings were obtained in the absence of synaptic blockers with an intracellular solution containing the following (in mm) 120 potassium gluconate, 10 KCl, 10 HEPES, 0.5 EGTA, 10 Na_2_-phosphocreatine, 2 Mg-ATP, and 0.3 Na-GTP (pH 7.2–7.3, 290 mOsm).

For voltage-clamp recordings of EPSCs, the potassium gluconate-based intracellular solution was used. Spontaneous EPSCs (sEPSCs) and evoked EPSCs were recorded at a holding potential of −70 mV in the presence of 100 μm picrotoxin (PTX: GABA-A receptor antagonist, Tocris Bioscience). Evoked NMDAR-mediated EPSCs (NMDAR-EPSCs) were recorded in ACSF with low MgCl_2_ (0.2 mm) at a holding potential of −60 mV and in the presence of 100 μm PTX and 25 μm of 6-cyano-7-nitroquinoxaline-2,3-dione (CNQX: AMPAR receptor antagonist, Abcam). For recordings of spontaneous IPSCs (sIPSCs) in CA1 pyramidal cells, a cesium-based intracellular solution containing (in mm) 120 CsMeSO_3_, 5 CsCl, 2 MgCl_2_, 10 HEPES, 0.5 EGTA, 10 Na_2_PO_4_, 2 ATP-Tris, 0.4 GTP-Tris, 0.1 spermine, and 2 QX314 (pH 7.2–7.3, and 280 mOsm) was used. Spontaneous IPSCs were recorded at a holding potential of 0 mV in the presence of 25 μm CNQX and 50 μm of D-(-)−2-amino-5-phosphonopentanoic acid (D-AP5; to block AMPA and NMDA receptors, respectively, Abcam).

Electrophysiological data were recorded using Multiclamp 700A/B amplifiers (Molecular Devices) and digitized at 20 kHz using Digidata 1440A and pClamp 10 (Molecular Devices). Recordings were low pass filtered at 2 kHz. Series resistance (15–25 mΩ) was monitored regularly during experiments and data were included if the holding current and series resistance were stable (changes <20% of initial value). Spontaneous EPSCs and sIPSCs were analyzed using Mini-Analysis 6.0.3 (Synaptosoft Inc.). For sEPSCs and sIPSCs, cells were recorded for a period of 5–10 min and 300 consecutive events were considered for analysis. For all experiments, data were acquired and analyzed blind to genotype.

### Electrophysiological data analysis

Electrophysiological data were analyzed with Clampfit 10.5 (Molecular Devices), Mini-Analysis 6.0.3 and GraphPad Prism 8 (GraphPad Software Inc.). Membrane properties of EGFP-positive interneurons were measured in current-clamp (Tricoire et al., 2011; Artinian et al., 2019). Resting membrane potential (RMP) was measured with the holding current I = 0 pA immediately after cell membrane rupture. Input resistance (R_in_) was measured using a linear regression of voltage deflections (±10–15 mV) in response to 800-ms-long current steps of 20 pA for fast-spiking EGFP-positive interneurons or 500-ms-long current steps of 10 pA for regular-spiking EGFP-positive interneurons from a holding potential of –60 mV. Rheobase was measured as the minimal current necessary to evoke an action potential. For FS-INs, the frequency-current (F-I) relationship for evoked firing was determined by injecting 800-ms somatic current steps of increasing amplitude (20-pA increments) to a maximum of 400 pA. For RS-INs, the F-I relationship was determined by injecting 500-ms somatic current steps of increasing amplitude (10-pA increments) to a maximum of 170 pA. The slope of the F-I relationship for each cell was calculated from a linear fit of the data for which the F-I relationship was approximatively linear.

AMPAR-mediated and NMDAR-mediated EPSCs were recorded in EGFP-positive interneurons using constant current pulse (50-μs duration) stimulation via an ACSF-filled theta-glass electrode, positioned ∼100 μm from the recorded cell in CA1 stratum oriens for FS-INs, or at the border between CA1 stratum oriens and the alveus for RS-INs. Minimally evoked EPSCs were obtained using minimal electrical stimulation at 0.5 Hz (with a success rate of ∼50%). Minimal EPSC amplitude (including successes and failures) and EPSC potency (including successes only) were calculated from 100–150 events. Paired pulse ratio of minimal EPSCs was calculated as a ratio of EPSC_2_ to EPSC_1_ (50-ms interstimulus interval). Synaptic currents input-output relationships were obtained by delivering stimulation current pulses of incremental intensity (50–600 μA) at 0.05 Hz, with five trials for each stimulation intensity. The slope (synaptic gain) and x-intercept (minimal stimulation intensity) of the linear regression of the input-output relationship were measured on averaged responses of individual cells. For short-term plasticity of EPSCs, five trains of 10 electrical stimuli at different frequencies (10, 20, and 50 Hz) were delivered at 20-s intervals. EPSC amplitude was normalized to the first EPSC amplitude. For EPSP-IPSP integration experiments, CA1 pyramidal cells were held at −60 mV and synaptic responses were obtained in current-clamp mode with electrical stimulation delivered by a concentric bipolar Pt/Ir electrode (FHC) placed in stratum oriens.

### Immunohistochemistry, confocal imaging, and neuronal cell counting

P60 mice were anaesthetized and transcardially perfused with saline followed by 4% paraformaldehyde (PFA) in phosphate buffer (PB; 0.1 m, pH 7.2). Brains were dissected out, postfixed overnight in 4% PFA at 4°C, and subsequently transferred to 30% sucrose in PBS (pH 7.2) for 48 h at 4°C. They were then embedded in moulds filled with optimal cutting temperature (OCT) Tissue Tek and frozen in a bath of 2-Methylbutane on a bed of dry ice and ethanol. Coronal sections (40 μm) were generated using a cryostat (Leica CM3050 S). Brain sections were blocked in 10% normal goat serum (NGS) with 1% Triton X-100 in 1× PBS for 2 h at RT. Sections were then incubated at 4°C for 48 h with the following primary antibodies diluted in 5% NGS + 0.1% Triton X-100 in 1× PBS: rabbit anti-PV (1:5000, Swant, catalog #PV27), mouse anti-NeuN (1:500, Millipore, catalog #MAB377), and chicken anti-GFP (1:500, Abcam, catalog #13970). Slices were rinsed in 0.1% Triton X-100 in PBS (three times) at RT between the primary and secondary antibody incubation. Subsequent incubation for 2 h at RT followed with secondary antibodies diluted in 5% NGS + 0.1% Triton X-100 in 1× PBS: Alexa 555-conjugated anti-rabbit IgG (1:500, Cell Signaling Technology, catalog #4413), Alexa 647-conjugated anti-mouse IgG (1:500, Cell Signaling Technology, catalog #4410) and Alexa 488-conjugated anti-chicken IgY (1:500, Abcam, catalog #ab150169). Finally, slices were rinsed in 1× PBS (three times) and mounted in Vectashield mounting medium (Vector).

Immunostained slices were imaged using the Leica SP8 confocal microscope at 20× (NA 0.75) at 1024 × 1024, zoom = 1, z-step = 1.5 μm. Confocal stacks of ∼20-μm depth were acquired from the CA1 region of the hippocampus from at least three coronal sections for each animal. At least 5 mice per genotype were used for the cell count analysis. All acquired images were analyzed in blind. Identical areas of 250 × 250 μm^2^ were analyzed semi automatically by either Neurolucida (MBF software) and or LAS X (Leica Application Suite X) software with two to three regions of interest (ROIs) per section (total = six to nine ROIs per animal). Total numbers of GFP^+^ cells, PV^+^ cells and both PV^+^GFP^+^ as well as PV^-^GFP^+^ cells were counted in the hippocampal CA1 area. Data are expressed as mean ± SEM and analyzed using Prism 8 (GraphPad Software Inc). Normality and equal variance were tested and differences between two groups were assessed with the Mann–Whitney rank sum test for not-normally distributed data.

### Whole genome sequencing

For whole genome sequencing (WGS), genomic DNA (gDNA) was extracted from the tail of a *Syngap1^flox/flox^* mouse, using the PureLink Genomic DNA Mini kit (ThermoFisher). Paired-end (2 × 150 bp) WGS was performed on a NovaSeq 6000 System (Illumina). Reads were first aligned onto the mouse reference genome GRCm38/mm10 using DRAGEN v.3.4.5 (Illumina). Variant calling was also done using DRAGEN v.3.4.5. Nonmatching sequences of over thirty nucleotides were identified from the list of called variants to locate the 34-bp loxP sequence insertions. The identity and position of these loxP sequences were confirmed with the Basic Local Assignment Search Tool (https://blast.ncbi.nlm.nih.gov/Blast.cgi) and by visual inspection using the Integrative Genomics Viewer tool version IGV_2.8.0 (http://www.broadinstitute.org/igv/).

### Characterization of the *Syngap1^flox^* allele by PCR

Various brain areas were isolated from *Nkx2.1-Syngap1^f/+^* and *Syngap1^f/+^* mice at 14 d of age [postnatal day (P)14], as well as from *Emx1-Syngap1^f/+^* and *Emx1-Syngap1^f/f^* mice at 24 d of age (P24). Genomic DNA was purified from these samples using either the PureLink Genomic DNA Mini kit (ThermoFisher Scientific) or the DNeasy Blood and Tissue kit (QIAGEN) according to the manufacturers’ instructions.

Cre-mediated inversion of *Syngap1* floxed exons 4–8 was revealed by PCR using two primers (pair B), which were designed to anneal with the same orientation to the same (-) gDNA strand of the gene so that amplification only becomes possible if the primers face each other on opposite strands as a result of the inversion. The forward primer (5′-GACTTGGATAGGCAGTCAGTCAG-3′), named B-i3Rv1, anneals 5′ to the loxP sequence in intron three on the (-) strand (directed toward exon 3) while the reverse primer (5′-ACTCTTCTATGGCTTTAGTGGCG-3′), named B-e9Rv2, anneals to the same strand 3′ to the loxP site in exon 9.

The absence of a recombination event within the *Syngap1^flox^* allele was demonstrated using primers (pair A) that anneal to opposite strands of exon 8 and 9, respectively: 5′-TTACCGGATGCTATGTGCAGTG-3′ (A-e8Fw1) and 5′-CTCGGAATATGAGGTGTTCCCG-3′ (A-e9Rv1).

### Statistical analysis

Data are expressed as mean ± SEM and analyzed using GraphPad Prism 8 (GraphPad Software Inc). Normality and equal variance were tested using the Shapiro–Wilk test and Levene median test, respectively. Differences between two groups were assessed with the Student’s *t* test for normally distributed data or with the Mann–Whitney rank sum test for not-normally distributed data. For multiple comparisons, mixed repeated-measures ANOVA were used for normally distributed data, and Friedman ANOVA was used for not-normally distributed data. Asterisks denote statistical significance as calculated by the specified statistical tests (*p <* *0.05, **p <* *0.01, ***p <* *0.001; ns indicates not significant). Sample size required to reach significance were determined with a power analysis with power = 0.8 and α = 0.05 (G*Power software). Details of statistical analyses are given in Extended Data Figure 1-1.

## Results

### Impairment of intrinsic and firing properties of hippocampal Nkx2.1 fast-spiking interneurons

SYNGAP1 is expressed in both excitatory and inhibitory neurons (Chen et al., 1998; Kim et al., 1998; Berryer et al., 2016). Hippocampal MGE-derived interneurons express the homeobox transcription factor Nkx2.1 and include somatostatin (SOM) and parvalbumin (PV) interneurons, as well as nitric oxide synthase (nNOS) expressing ivy and neurogliaform cells (Tricoire et al., 2011; Pelkey et al., 2017). To test whether conditional heterozygous knock-out (KO) of *Syngap1* in MGE-derived inhibitory cells impairs interneuron function, we used *Nkx2.1-Syngap1^f/+^* mice and *Nkx2.1-Syngap1^+/+^* mice. The Cre-dependent expression of RCE (Xu et al., 2008) was included to identify EGFP^+^ Nkx2.1-expressing (Nkx2.1^+^) cells in hippocampal slices of adult mice.

**Figure 1.**
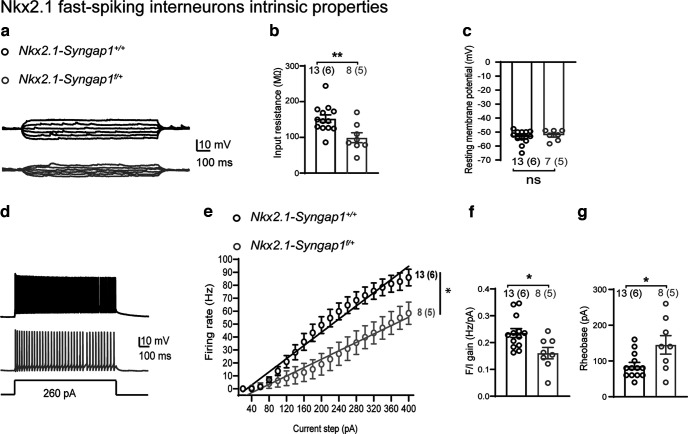
Reduction of input resistance and evoked firing in hippocampal Nkx2.1 fast-spiking interneurons. ***a***, Representative traces of membrane response during somatic current injection (±60-pA steps, 800-ms duration) in CA1 Nkx2.1^+^ FS-INs from *Nkx2.1-Syngap1^+/+^* (black) and *Nkx2.1-Syngap1^f/+^* (gray) mice. ***b***, Summary plot showing reduced cell input resistance of Nkx2.1^+^ FS-INs from *Nkx2.1-Syngap1^f/+^* (*n* = 8 cells, 5 mice) relative to *Nkx2.1-Syngap1^+/+^* mice (*n* = 13 cells, 6 mice). Unpaired *t* test: **p = 0.0063. ***c***, Summary graph showing unchanged resting membrane potential of Nkx2.1^+^ FS-INs from *Nkx2.1-Syngap1^f/+^* (*n* = 7 cells, 5 mice) compared with *Nkx2.1-Syngap1^+/+^* mice (*n* = 13 cells, 6 mice). Unpaired *t* test: *p *=* *0.54. ***d***, Representative traces of the action potential train recorded from Nkx2.1^+^ FS-INs from *Nkx2.1-Syngap1^+/+^* and *Nkx2.1-Syngap1^f/+^* mice in response to 260-pA current injection step (800-ms duration). ***e***, Summary graph with linear fit of the frequency-current (F/I) relationships showing reduced firing in response to somatic depolarization in Nkx2.1^+^ FS-INs from *Nkx2.1-Syngap1^f/+^* mice (*n* = 8 cells, 5 mice) relative to Nkx2.1^+^ FS-INs from *Nkx2.1-Syngap1^+/+^* (*n* = 13 cells, 6 mice). Mixed repeated-measures ANOVA: *F*_(1,19 genotype)_ = 7.272, *p =* *0.0143. ***f***, ***g*,** Summary graphs showing reduced slope of F-I relationships (***f***) and increased rheobase (***g***) in Nkx2.1^+^ FS-INs of mutant mice (*n* = 8 cells, 5 mice) relative to control littermates (*n* = 13 cells, 6 mice). Unpaired *t* tests: *p =* *0.012 (***f***), *p = 0.0217 (***g***). Details of statistical analyses in this and following figures are given in Extended Data Figure 1-1.

First, we performed whole-cell current-clamp recordings from Nkx2.1^+^ interneurons located in the CA1 stratum pyramidale or at the stratum oriens/stratum pyramidale border to target fast-spiking interneurons (FS-INs; likely corresponding to parvalbumin expressing interneurons). Nkx2.1^+^ FS-INs were physiologically-identified by their action potential firing at high frequency without adaptation, short membrane time constant and large after-hyperpolarization (Du et al., 1996). We found that Nkx2.1^+^ FS-INs display similar resting membrane potential (RMP) but reduced input resistance (R_in_) in *Nkx2.1-Syngap1^f/+^* mice compared with control *Nkx2.1-Syngap1^+/+^* mice (Fig. 1*a–c*). Also, we found that Nkx2.1^+^ FS-INs fire less action potentials in response to somatic depolarization in *Nkx2.1-Syngap1^f/+^* mice compared with control mice (Fig. 1*d*), showing a decrease in the frequency-current (F/I) relationship (Fig. 1*e*,*f*). The rheobase current necessary to elicit a single action potential in Nkx2.1^+^ FS-INs was also elevated in *Nkx2.1-Syngap1^f/+^* mice compared with control mice (Fig. 1*g*). Thus, *Syngap1* decrease in MGE-derived interneurons impairs basic intrinsic and firing properties of hippocampal Nkx2.1^+^ FS-INs.

10.1523/ENEURO.0475-22.2023.f1-1 Extended Data Figure 1-1 Statistical summary table. Download Figure 1-1, DOC file.

### Facilitation of AMPAR-mediated synaptic excitation of Nkx2.1 fast-spiking interneurons

**Figure 2.**
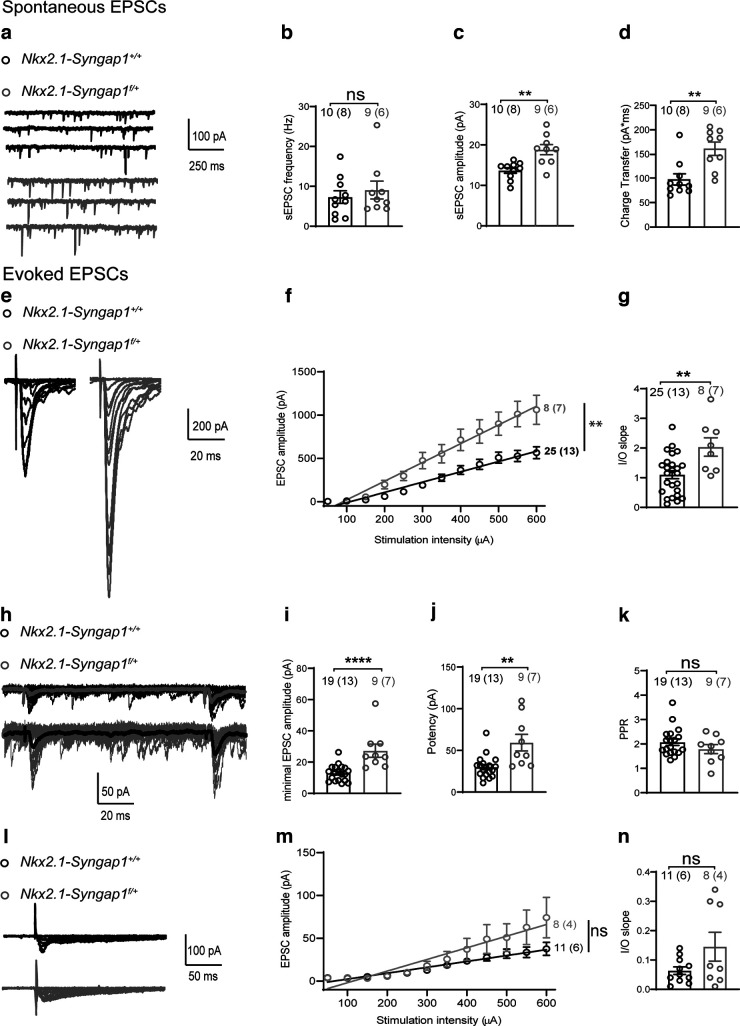
Increase in AMPAR-mediated but not NMDAR-mediated excitatory synaptic transmission in Nkx2.1 fast-spiking interneurons. ***a***, Representative traces of AMPAR-mediated sEPSCs in Nkx2.1^+^ FS-INs from *Nkx2.1-Syngap1^+/+^* (black) and *Nkx2.1-Syngap1^f/+^* (gray). ***b***, Summary plot showing unchanged sEPSC frequency in *Nkx2.1-Syngap1^f/+^* mice (*n* = 9 cells, 6 mice) relative to *Nkx2.1-Syngap1^+/+^* mice (*n* = 10 cells, 8 mice; Mann–Whitney test, *p *=* *0.6607). ***c***, ***d***, Summary plots showing increased amplitude (***c***) and charge transfer (***d***) of sEPSCs in *Nkx2.1-Syngap1^f/+^* mice (*n* = 9 cells, 6 mice) relative to *Nkx2.1-Syngap1^+/+^* mice (*n* = 10 cells, 8 mice) unpaired *t* tests: (***c***) **p =* *0.0017, (***d***) **p =* *0.0025). ***e****–****g***, Representative traces of families of AMPAR-mediated EPSCs evoked by electrical stimulation of increasing intensity (***e***), summary graphs with linear fit of the input-output (I/O) relationship of EPSC amplitude versus stimulation intensity (***f***) and of the slope of the I/O relationship (***g***), showing that the input–output function of evoked EPSCs was increased Nkx2.1^+^ FS-INs from *Nkx2.1-Syngap1^f/+^* mice (*n* = 8 cells, 7 mice) relative to *Nkx2.1-Syngap1^+/+^* mice (*n* = 25 cells, 13 mice). Mixed repeated-measures ANOVA (***f***): *F*_(1,31 genotype)_ = 11.60, **p =* *0.0018. Unpaired *t* test (***g***): **p =* *0.0036. ***h–k***, Representative traces of AMPAR-mediated EPSCs evoked by minimal stimulation (***h***, with mean EPSC superimposed), and summary plots of EPSC amplitude (***i***, mean of successes + failures), potency (***j***, mean of successes only), and paired-pulse ratio (***k***, PPR), showing increased EPSC amplitude (Mann–Whitney test: ****p < 0.0001) and potency (Mann–Whitney test: **p = 0.0020) without change in PPR (unpaired *t* test: *p *=* *0.2327) in *Nkx2.1-Syngap1^f/+^* mice (*n* = 9 cells, 7 mice) relative to *Nkx2.1-Syngap1^+/+^* mice (*n* = 19 cells, 13 mice). ***l–n***, Representative traces of families of NMDAR-mediated EPSCs evoked by electrical stimulation of increasing intensity (***l***), summary graph with linear fit of the input-output (I/O) relationship of EPSC amplitude versus stimulation intensity (***m***) and of the slope of the I/O relationship (***n***) showing unchanged I/O relationship of NMDAR-mediated EPSC in *Nkx2.1-Syngap1^f/+^* mice (*n* = 8 cells, 4 mice) relative to *Nkx2.1-Syngap1^+/+^* mice (*n* = 11 cells, 6 mice). Mixed repeated-measures ANOVA (***m***): *F*_(1,17 genotype)_ = 1.900, *p *=* *0.1859. Unpaired *t* test (***n***): *p* = 0.0814. Power analysis (***n***) sample size required to reach significance = 54 cells.

Given that AMPAR-mediated synaptic transmission is regulated by SYNGAP1 in excitatory neurons (Clement et al., 2012; Wang et al., 2013; Aceti et al., 2015; Araki et al., 2015; Walkup et al., 2016), we explored whether *Syngap1* decrease in MGE-derived interneurons affects AMPAR-mediated synaptic inputs to Nkx2.1^+^ interneurons. First, we examined spontaneous EPSCs (sEPSCs) in Nkx2.1^+^ FS-INs (Fig. 2*a–d*). We found that the sEPSC amplitude and charge transfer (area under sEPSC) were increased in *Nkx2.1-Syngap1^f/+^* mice relative to *Nkx2.1-Syngap1^+/+^*, but the frequency of sEPSCs was unchanged.

Next, we recorded AMPAR-mediated EPSC evoked in FS-INs by electrical stimulation in the stratum oriens. We observed an increase in the input-output (I/O) relationship (Fig. 2*e*,*f*) and slope of I/O curve (Fig. 2*g*) of AMPAR-mediated EPSCs in Nkx2.1^+^ FS-INs from *Nkx2.1-Syngap1^f/+^* mice relative to *Nkx2.1-Syngap1^+/+^* mice. We examined also whether EPSCs evoked by activation of putative single excitatory fiber were affected by recording AMPAR-mediated EPSCs evoked in Nkx2.1^+^ FS-INs by minimal extracellular stimulation. Minimally-evoked EPSC amplitude (average of all responses, successes + failures) and potency (average of all successes only) were increased in *Nkx2.1-Syngap1^f/+^* mice compared with control littermates (Fig. 2*h–j*). Paired-pulse facilitation was unchanged (Fig. 2*k*), suggesting that the increase in synaptic excitation onto Nkx2.1^+^ FS-INs in *Nkx2.1-Syngap1^f/+^* mice was likely because of postsynaptic mechanisms. These results indicate that *Syngap1* decrease in MGE-derived interneurons facilitates AMPAR-mediated synaptic input onto Nkx2.1^+^ FS-INs.

Next, we assessed the effect of *Syngap1* decrease in MGE-derived inhibitory cells on NMDAR-mediated EPSCs in Nkx2.1^+^ FS-INs. To do this, electrically-evoked NMDAR-EPSCs were isolated pharmacologically by blocking GABA_A_R-mediated and AMPAR-mediated synaptic responses (100 μm PTX and 20 μm CNQX, respectively) and reducing extracellular Mg^2+^. We found that NMDAR-mediated EPSCs in Nkx2.1^+^ FS-INs were similar in *Nkx2.1-Syngap1^f/+^* and *Nkx2.1-Syngap1^+/+^* mice. The NMDAR-mediated EPSC amplitude I-O relationship and I-O slope were unchanged (Fig. 2*l–n*). In line with its role in excitatory pyramidal neurons, these results suggest that *Syngap1* decrease in Nkx2.1^+^ FS-INs specifically facilitates AMPAR-mediated but not NMDAR-mediated synaptic transmission.

### Impairment of short-term dynamics of AMPAR-mediated synaptic excitation of Nkx2.1 fast-spiking interneurons

**Figure 3.**
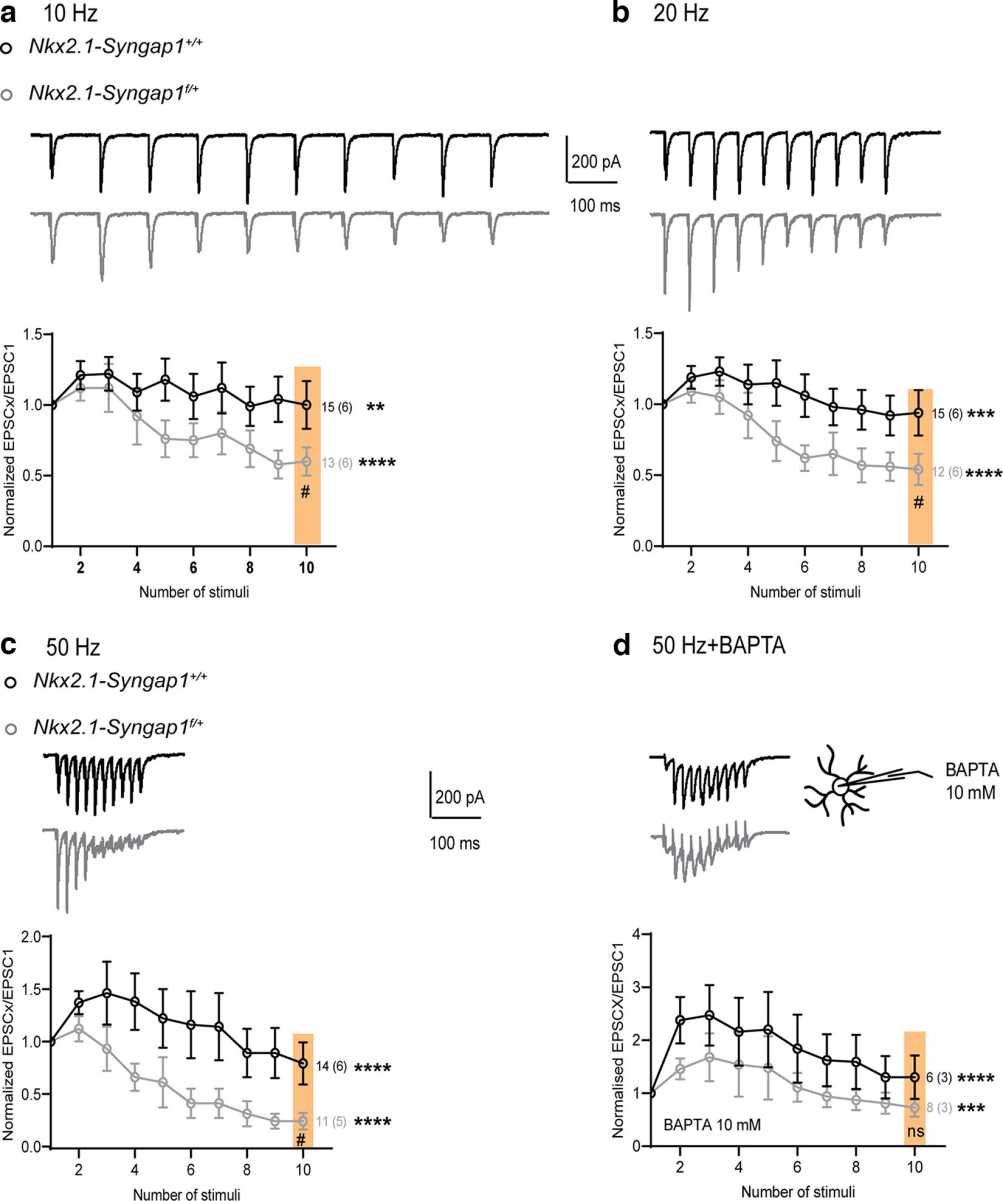
Impairment of short-term facilitation of EPSCs in Nkx2.1 fast-spiking interneurons. ***a****–****c***, Representative traces of EPSCs (top) and summary graphs (bottom) of normalized EPSC amplitude during stimulation trains given at 10 (***a***), 20 (***b***), and 50 (***c***) Hz, showing impairment of short-term facilitation of EPSCs in Nkx2.1^+^ FS-INs from *Nkx2.1-Syngap1^f/+^* mice relative to *Nkx2.1-Syngap1^+/+^* mice. 10 Hz (***a***): Friedman ANOVAs, *Nkx2.1-Syngap1^+/+^* (*n* = 15 cells, 6 mice) **p = 0.003, *Nkx2.1-Syngap1^f/+^* (*n* = 13 cells, 6 mice) ****p <* *0.0001; Mann–Whitney test stim 10 (color shading), #*p *=* *0.04. 20 Hz (***b***): Friedman ANOVAs, *Nkx2.1-Syngap1^+/+^* (*n* = 15 cells, 6 mice) ***p = 0.0002, *Nkx2.1-Syngap1^f/+^* (*n* = 12 cells, 6 mice) ****p <* *0.0001; Mann–Whitney test stim 10 (color shading), #*p *=* *0.02. 50 Hz (***c***): Friedman ANOVAs, *Nkx2.1-Syngap1^+/+^* (*n* = 14 cells, 6 mice) ****p <* *0.0001, *Nkx2.1-Syngap1^f/+^* (*n* = 11 cells, 5 mice) ****p <* *0.0001; Mann–Whitney test stim 10 (color shading), #*p *=* *0.01. ***d***, Representative traces of EPSCs (top) and summary plot (bottom) of normalized EPSC amplitudes in response to repetitive stimulation at 50 Hz in the presence 10 mm BAPTA in the intracellular solution, showing rescue of short-term facilitation in Nkx2.1^+^ FS-INs from *Nkx2.1-Syngap1^f/+^* mice. Friedman ANOVAs, *Nkx2.1-Syngap1^+/+^* (*n* = 6 cells, 3 mice) ****p <* *0.0001, *Nkx2.1-Syngap1^f/+^* (*n* = 8 cells, 3 mice) ***p =* *0.0005; Mann–Whitney test stim 10 (color shading), *p *=* *0.49, ns indicates not significant.

Since *Syngap1* decrease in MGE-derived interneurons affects synaptic excitation of Nkx2.1^+^ FS-INs, we examined whether short-term dynamics of these synaptic responses were altered. We analyzed short-term plasticity of EPSCs in Nkx2.1^+^ FS-INs during a train of 10 stimuli given at frequencies of 10, 20, and 50 Hz (Fig. 3*a–c*) by an extracellular stimulation pipette in stratum oriens. We found that in control *Nkx2.1-Syngap1^+/+^* mice, EPSCs showed short-term facilitation during the first few stimuli of the trains, which recovered to baseline level at the end of the train, for all stimulation frequencies. In contrast, in *Nkx2.1-Syngap1^f/+^* mice, EPSCs displayed short-term depression that reached a plateau during the stimulation trains, indicating that short-term dynamics of AMPAR-mediated synaptic excitation of Nkx2.1^+^ FS-INs is deficient after conditional *Syngap1* decrease in these cells.

Given the role of postsynaptic Ca^2+^-dependent signaling in the regulation of AMPAR-mediated synaptic excitation in principal cells (Borgdorff and Choquet, 2002; Heine et al., 2008; Opazo et al., 2010; Constals et al., 2015) and FS-INs (Camiré et al., 2012), we tested whether preventing postsynaptic Ca^2+^ rises during high frequency stimulation could rescue the deficit in EPSC short-term dynamics in *Nkx2.1-Syngap1^f/+^* mice. We included the fast Ca^2+^ chelator BAPTA (10 mm) in the intracellular solution during whole cell recordings from Nkx2.1^+^ FS-INs and measured EPSC short-term plasticity during a 50-Hz stimulation train (Fig. 3*d*). We found that in the presence of intracellular BAPTA, EPSCs from *Nkx2.1-Syngap1^f/+^* mice did not show depression, but instead showed facilitation during the first few stimuli and recovered to baseline level at the end of the train, similarly to EPSCs from *Nkx2.1-Syngap1^+/+^* mice (Fig. 3*c*,*d*). In the presence of intracellular BAPTA, EPSCs from *Nkx2.1-Syngap1^+/+^* mice displayed stronger facilitation. Thus, reducing postsynaptic Ca^2+^ rise rescued the deficit in short-term facilitation of AMPAR-mediated excitatory synapses in Nkx2.1^+^ FS-INs from *Nkx2.1-Syngap1^f/+^* mice, suggesting that the deficit in short-term dynamics caused by conditional *Syngap1* decrease in these cells involve postsynaptic Ca^2+^-dependent signaling.

### Membrane and synaptic properties of Nkx2.1 regular-spiking interneurons

**Figure 4.**
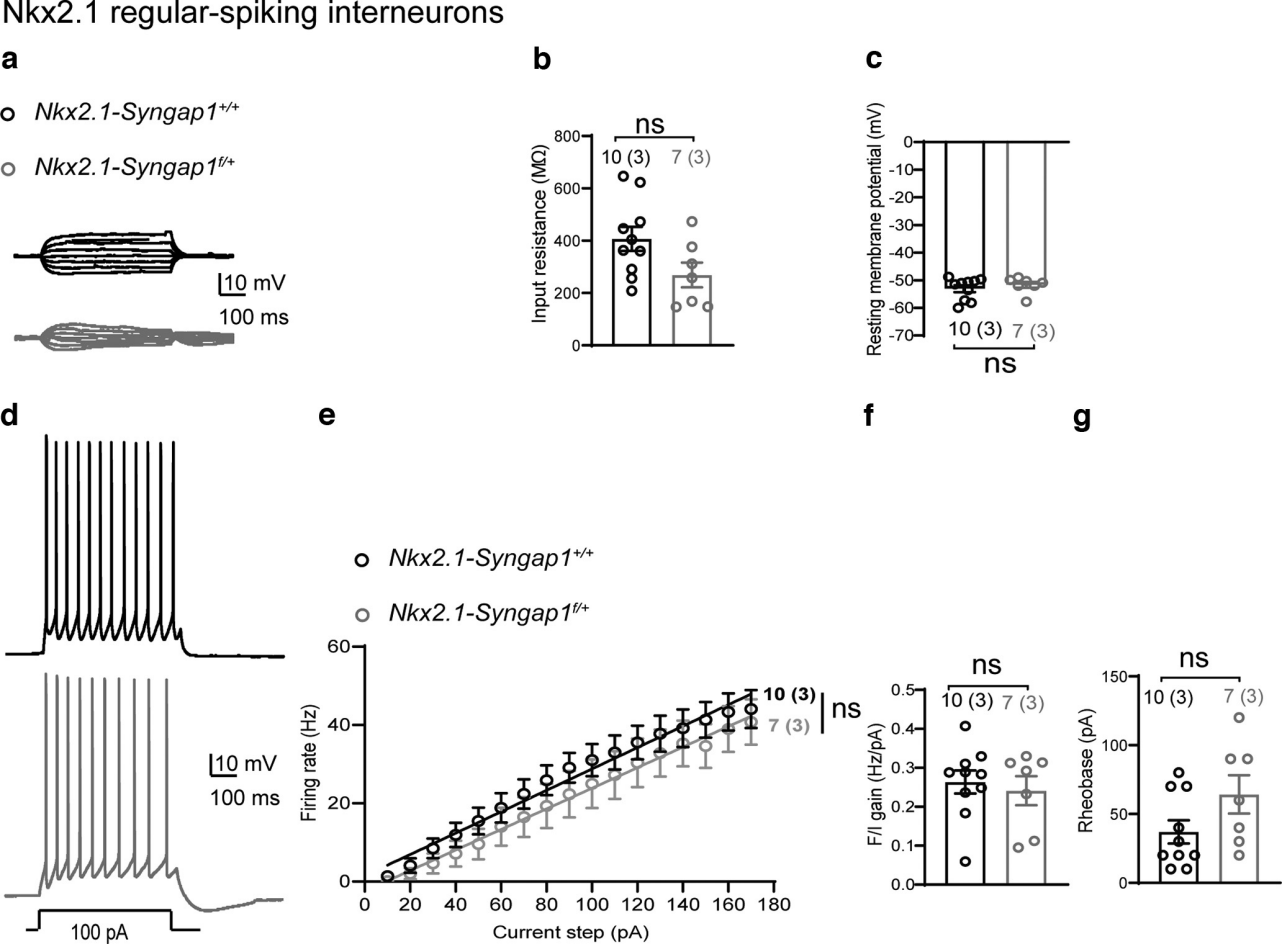
Intact membrane and firing properties of Nkx2.1 regular-spiking interneurons. ***a***, Representative traces of membrane responses of Nkx2.1^+^ RS-INs to current injection (±30 pA, 10-pA steps, 500 ms). ***b***, Summary graph showing unchanged cell input resistance of Nkx2.1^+^ RS-INs from *Nkx2.1-Syngap1^f/+^* mice (*n* = 7 cells, 3 mice) compared with *Nkx2.1-Syngap1^+/+^* mice (*n* = 10 cells, 3 mice). Mann–Whitney test: *p* = 0.06. ***c***, Summary graph indicating similar resting membrane potential of Nkx2.1^+^ RS-INs in *Nkx2.1-Syngap1^+/+^* (*n* = 10 cells, 3 mice) and *Nkx2.1-Syngap1^f/+^* mice (*n* = 7 cells, 3 mice). Mann–Whitney test: *p *=* *0.6175. ***d***, Representative traces showing regular-spiking pattern of action potentials in response to somatic current injection. ***e***, Summary graph with linear fit of the frequency-current (F/I) relationships showing unchanged firing of Nkx2.1^+^ RS-INs from *Nkx2.1-Syngap1^f/+^* mice (*n* = 7 cells, 3 mice) relative to *Nkx2.1-Syngap1^+/+^* mice (*n* = 10 cells, 3 mice). Mixed repeated-measures ANOVA: *F*_(1,15 genotype)_ = 0.7545, *p *=* *0.3987. ***f***, ***g***, Summary plots of the slope of the evoked firing frequency-current relationship (F/I gain; ***f***) and rheobase current (***g***), showing unchanged firing properties in RS-INs from *Nkx2.1-Syngap1^f/+^* mice (*n* = 7 cells, 3 mice) relative to *Nkx2.1-Syngap1^+/+^* mice (*n* = 10 cells, 3 mice). Unpaired *t* tests: *p *=* *0.64 in ***f***, *p = *0.09 in ***g***; ns indicates not significant. Power analysis (***g***) sample size required to reach significance = 48 cells.

MGE-derived neurons account for 60% of CA1 GABAergic cells and include different types of interneurons such as SOM and PV interneurons, as well as nitric oxide synthase (nNOS)-expressing ivy and neurogliaform interneurons (Tricoire et al., 2011; Pelkey et al., 2017). PV and SOM interneurons represent ∼70% of MGE-derived interneurons (Tricoire et al., 2011; Pelkey et al., 2017). To examine whether *Syngap1* haploinsufficiency in MGE-derived interneurons result in interneuron subtype specific effects, we targeted putative SOM interneurons by recording from Nkx2.1^+^ regular-spiking (RS) interneurons (RS-INs) located at the CA1 stratum oriens/alveus border in *Nkx2.1-Syngap1^f/+^* and *Nkx2.1-Syngap1^+/+^* mice. In current-clamp recordings (Fig. 4*a–c*), we found intact basic properties of RS-INs. Cell input resistance (Fig. 4*b*) and resting membrane potential (Fig. 4*c*) were unchanged in *Nkx2.1-Syngap1^f/+^* mice relative to *Nkx2.1-Syngap1^+/+^* mice. Similarly, the evoked firing properties were unaffected in RS-INs (Fig. 4*d–f*). The evoked firing frequency-current relationship (F/I gain; Fig. 4*e*,*f*) and the rheobase current (Fig. 4*g*) were similar in *Nkx2.1-Syngap1^f/+^* mice and *Nkx2.1-Syngap1^+/+^* mice. These data indicate that *Syngap1* decrease in MGE-derived interneurons has cell type-specific effects on basic membrane and firing properties of Nkx2.1 FS-INs but not RS-INs.

**Figure 5.**
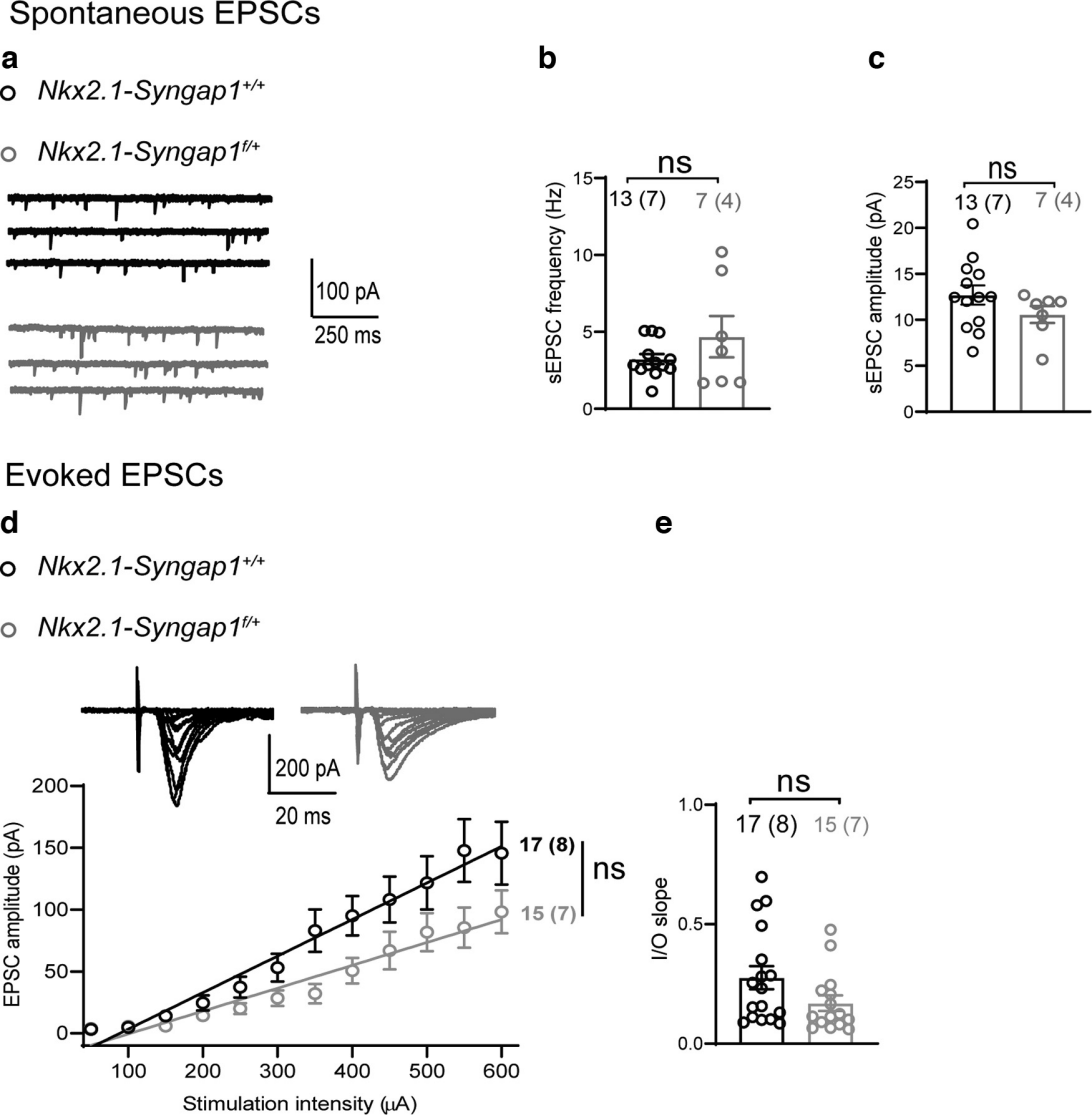
Intact spontaneous and evoked excitatory synaptic transmission in Nkx2.1 regular-spiking interneurons. ***a***, Representative traces of sEPSCs recorded in Nkx2.1^+^ RS-INs. ***b***, ***c***, Summary bar graphs showing unchanged sEPSC frequency (***b***, unpaired *t* test with Welch’s correction: *p *=* *0.3277) and amplitude (***c***, unpaired *t* test with Welch’s correction: *p *=* *0.1415) in Nkx2.1^+^ RS-INs from *Nkx2.1-Syngap1^f/+^* mice (*n* = 7 cells, 4 mice) compared with *Nkx2.1-Syngap1^+/+^* mice (*n* = 13 cells, 7 mice). Power analysis (***b***, ***c***) sample size required to reach significance = 106 cells (***b***) and 118 cells (***c***). ***d***, ***e***, Representative families of traces of EPSCs evoked by electrical stimulation of increasing intensity (***d***, top), and summary graphs of the input-output (I/O) relationship with linear fit of EPSC amplitude versus stimulation intensity (***d***, bottom) and of the slope of the I/O relationship (***e***), showing unchanged input-output function of evoked EPSCs (***d***, mixed repeated-measures ANOVA**:** *F*_(1,30 genotype)_ = 3.786,*p *=* *0.0611) and I/O slope (***e***, unpaired *t* test, *p *=* *0.08) in Nkx2.1^+^ RS-INs from *Nkx2.1-Syngap1^f/+^* mice (*n* = 15 cells, 7 mice) compared with *Nkx2.1-Syngap1^+/+^* mice (*n* = 17 cells, 8 mice). Power analysis (***e***) sample size required to reach significance = 104 cells. ns indicates not significant.

Next, we examined whether *Syngap1* decrease in MGE-derived interneurons produced cell type-specific effects on synaptic excitation. First, we measured spontaneous EPSCs in Nkx2.1^+^ RS-INs. We found that both sEPSC frequency and amplitude were similar (Fig. 5*a–c*), indicating unaffected spontaneous excitatory synaptic activity in *Nkx2.1-Syngap1^f/+^* relative to *Nkx2.1-Syngap1^+/+^* mice. Second, we stimulated excitatory inputs onto Nkx2.1^+^ RS-INs through an extracellular stimulation electrode positioned at the oriens/alveus border and recorded evoked EPSCs. We found that the input-output relationship of evoked EPSCs (Fig. 5*d*) and the slope of the I/O relationship (Fig. 5*e*) were unchanged in Nkx2.1^+^ RS-INs from *Nkx2.1-Syngap1^f/+^* mice compared with *Nkx2.1-Syngap1^+/+^* mice. These results suggest that *Syngap1* decrease in MGE-derived interneurons does not affect evoked excitatory synaptic responses in Nkx2.1^+^ RS-INs, indicating cell type-specific effects on excitatory synaptic transmission in Nkx2.1 RS-INs and FS-INs.

**Figure 6.**
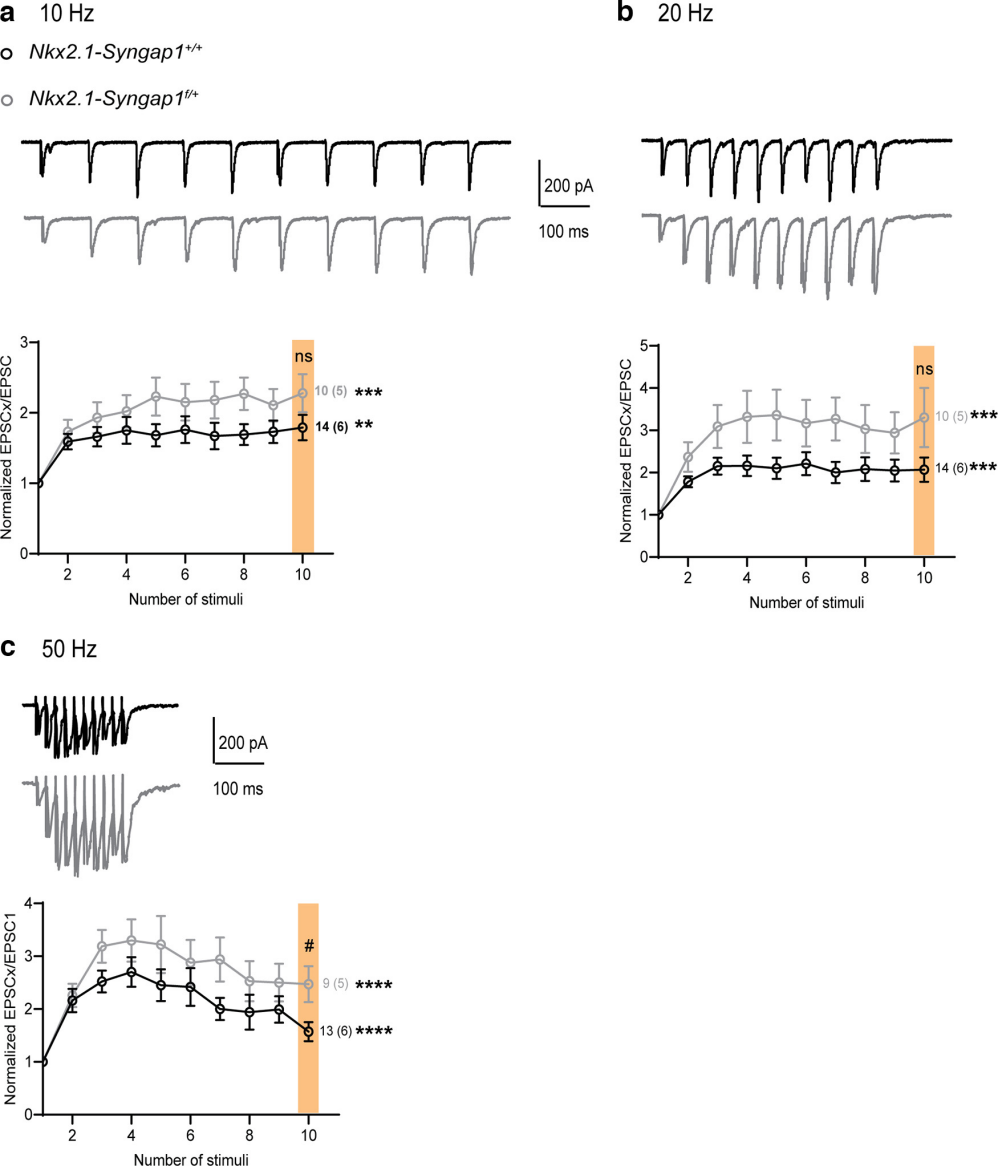
Short-term facilitation of EPSCs in Nkx2.1 regular-spiking interneurons. ***a–c***, Representative traces of EPSCs (top) and summary graphs (bottom) of normalized EPSC amplitude during stimulation trains given at 10 (***a***), 20 (***b***), and 50 (***c***) Hz, showing mostly intact short-term facilitation of EPSCs in Nkx2.1^+^ RS-INs from *Nkx2.1-Syngap1^f/+^* mice relative to *Nkx2.1-Syngap1^+/+^* mice. 10 Hz (***a***): Friedman ANOVAs, *Nkx2.1-Syngap1^+/+^* (*n* = 14 cells, 6 mice) **p = 0.0020, *Nkx2.1-Syngap1^f/+^* (*n* = 10 cells, 5 mice) ***p = 0.0002; Mann–Whitney test stim 10 (color shading), *p *=* *0.15. 20 Hz (***b***): Friedman ANOVAs, *Nkx2.1-Syngap1^+/+^* (*n* = 14 cells, 6 mice) ***p = 0.0002, *Nkx2.1-Syngap1^f/+^* (*n* = 10 cells, 5 mice) ***p = 0.0002; Mann–Whitney test stim 10 (color shading), *p *=* *0.19. 50 Hz (***c***): Friedman ANOVAs, *Nkx2.1-Syngap1^+/+^* (*n* = 13 cells, 6 mice) ****p <* *0.0001, *Nkx2.1-Syngap1^f/+^* (*n* = 9 cells, 5 mice) ****p <* *0.0001; Mann–Whitney test stim 10 (color shading), #*p *=* *0.03. ns indicates not significant.

Next, we examined the effects of *Syngap1* decrease in MGE-derived interneurons on the short-term dynamics of synaptic excitation of Nkx2.1^+^ RS-INs. We compared short-term plasticity of evoked EPSCs during brief trains at different frequencies (10 stimuli at 10, 20, and 50 Hz, Fig. 6*a–c*). In Nkx2.1^+^ RS-INs of control *Nkx2.1-Syngap1^+/+^* mice, EPSCs amplitude increased during the first few stimulations of the train and then maintained a plateau of facilitation for all stimulation frequencies (Fig. 6*a–c*). In *Nkx2.1-Syngap1^f/+^* mice, EPSCs in Nkx2.1^+^ RS-INs showed similar facilitation, except for a small decrease in facilitation with 50-Hz stimulation (Fig. 6*c*), compared with *Nkx2.1-Syngap1^+/+^* mice, suggesting mostly unchanged short-term plasticity in Nkx2.1^+^ RS-INs, and a further indication of cell type-specific effects after *Syngap1* decrease in MGE-derived interneurons.

### Impairment of pyramidal cell synaptic inhibition and facilitation of excitatory postsynaptic integration

**Figure 7.**
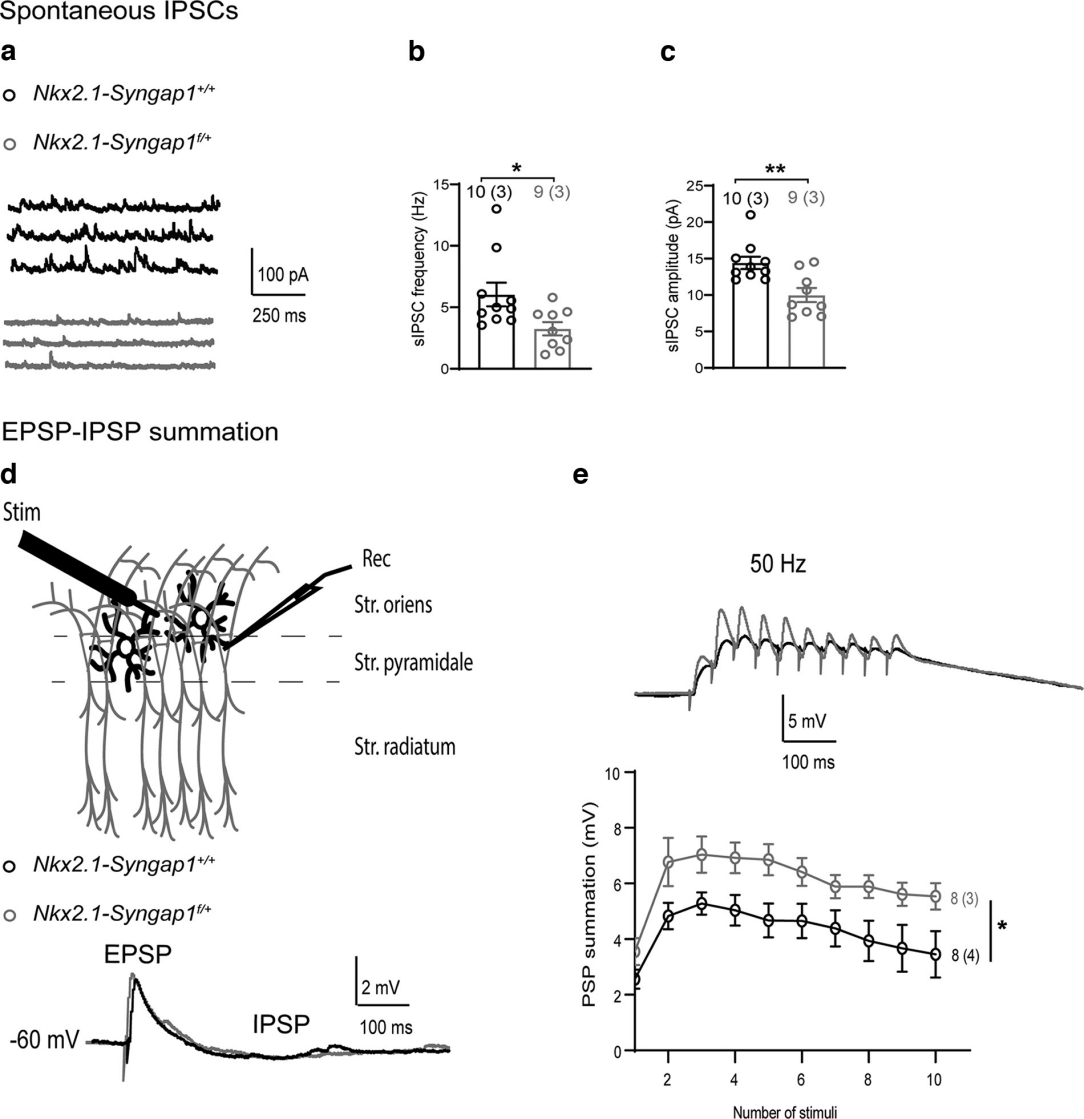
Reduction of synaptic inhibition and facilitated summation of excitatory synaptic responses in pyramidal cells. ***a–c***, Representative traces of spontaneous IPSCs (sIPSCs) in CA1 pyramidal cells (***a***) with summary plots of sIPSC frequency (***b***) and amplitude (***c***) showing reduction of frequency and amplitude from *Nkx2.1-Syngap1^f/+^* mice (*n* = 9 cells, 3 mice) compared with *Nkx2.1-Syngap1^+/+^* (*n* = 10 cells, 3 mice). Mann–Whitney tests, *p =* *0.022, **p =* *0.005. ***d***, Diagram of electrode positions for EPSP-IPSP summation experiments (top) and representative traces of compound postsynaptic potentials (EPSP-IPSP) recorded in pyramidal cells at −60 mV. ***e***, Representative traces (top) and summary graph (bottom) of response summation (EPSP-IPSPs) during repetitive stimulation, showing facilitated postsynaptic summation of EPSPs in *Nkx2.1-Syngap1^f/+^* mice (*n* = 8 cells, 3 mice) compared with *Nkx2.1-Syngap1^+/+^* mice (*n* = 8 cells, 4 mice). Two-way repeated measure ANOVA *F*_(1,14)_ = 6.105, *p =* *0.027.

Since *Syngap1* decrease in MGE-derived interneurons affects intrinsic and synaptic properties of FS-INs, we examined the effect on pyramidal cell synaptic inhibition and postsynaptic integration. First, we recorded spontaneous IPSCs (sIPSCs) in CA1 pyramidal neurons and found that sIPSC frequency and amplitude were reduced in *Nkx2.1-Syngap1^f/+^* mice relative to *Nkx2.1-Syngap1^+/+^* mice (Fig. 7*a–c*). These results are consistent with the previous report of decreased miniature IPSCs (Berryer et al., 2016), indicating a deficit in synaptic inhibition of pyramidal cells.

Next, we assessed what is the net effect of the global changes in interneuron functions on postsynaptic integration in pyramidal cells and examined the summation of EPSPs in pyramidal neurons. To determine this, we first recorded postsynaptic potentials (PSPs) in pyramidal cells in current-clamp mode at a holding potential of −60 mV in response to electric stimulation delivered through a stimulation electrode in the stratum oriens (Fig. 7*d*, top). After adjusting the stimulation intensity to get PSPs of ≈2 mV amplitude (compound EPSP-IPSP; Fig. 7*d*, bottom), we delivered 10 stimuli at 50 Hz and measured EPSPs summation in pyramidal neurons (Fig. 7*e*). We found that the amplitude of EPSPs in pyramidal cells from both genotypes increase during the first few stimuli of the train, reaching a plateau level from the third stimulation of the train. Interestingly, in pyramidal cells from *Nkx2.1-Syngap1^f/+^* mice, the summation of EPSPs was facilitated relative to *Nkx2.1-Syngap1^+/+^* mice (Fig. 7*e*). Thus, *Syngap1* decrease in MGE-derived interneurons results in facilitation of EPSP summation in CA1 pyramidal cells, consistent with a decrease in synaptic inhibition.

### Decreased number of MGE-derived cells in the hippocampus of *Nkx2.1-Syngap1^f/+^* mice

**Figure 8.**
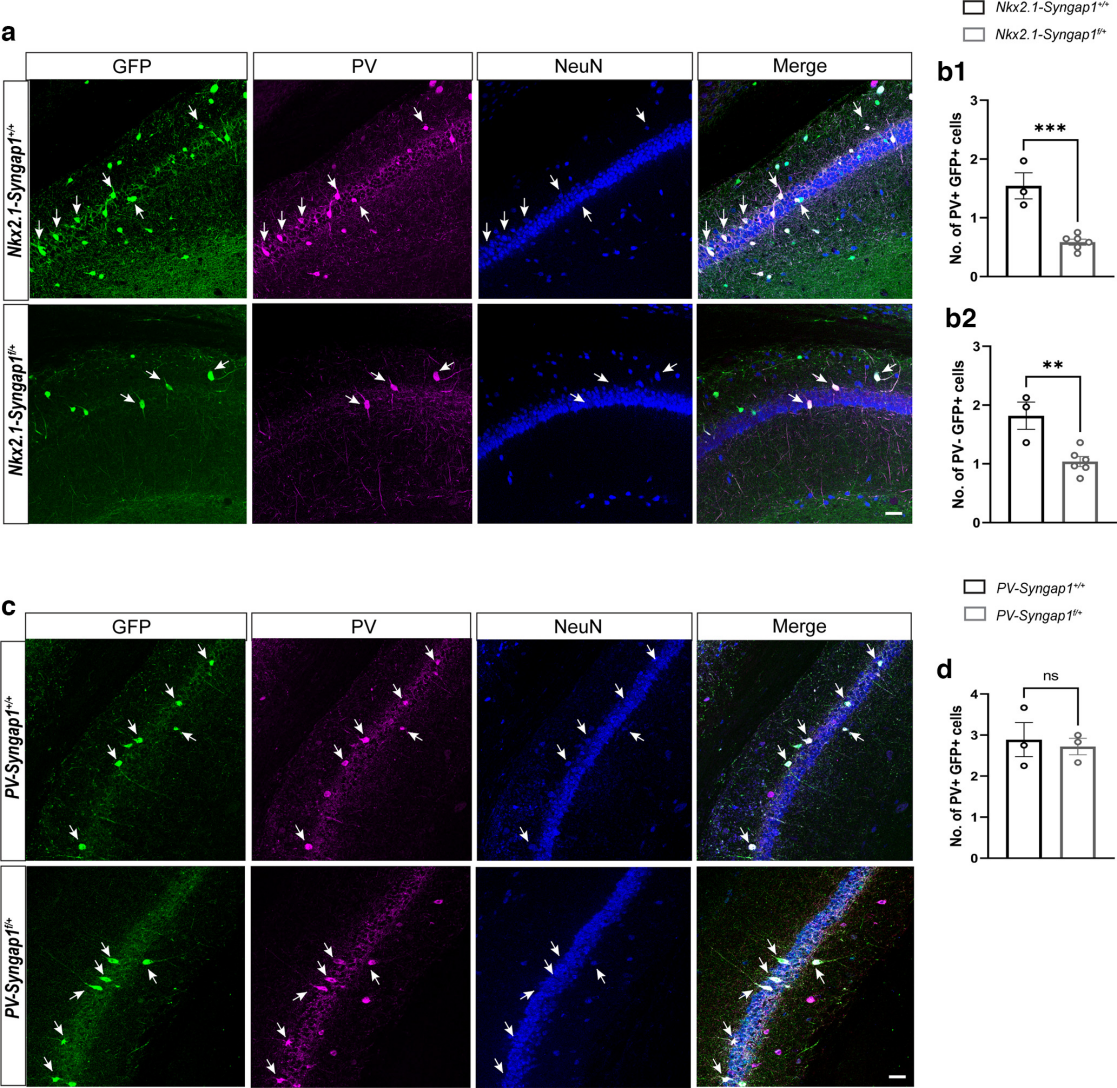
Number of PV interneurons in the CA1 area of the hippocampus of *PV-Syngap1^f/+^* and *Nkx2.1-Syngap1^f/+^* mice. ***a***, Confocal images of GFP^+^PV^+^ cells (arrows) in the hippocampal CA1 from both *Nkx2.1-Syngap1^+/+^* (*n* = 3) and *Nkx2.1-Syngap1^f/+^* (*n* = 6) mice show significant decrease in the numbers of both GFP^+^PV^+^ cells (***b1***; Mann–Whitney test: *p* = 0.0006) and GFP^+^PV^−^ cells (***b2***; Mann–Whitney test: *p* = 0.0054) **c.** Confocal images of GFP^+^PV^+^ cells (arrows) in the hippocampal CA1 from both *PV-Syngap1^+/+^* (*n* = 3) and *PV-Syngap1^f/+^* (*n* = 3) showed no change in the numbers of GFP^+^PV^+^ cells (***d***; Mann–Whitney test: *p* = 0.7358). See Extended Data Figure 8-1, number of PV interneurons in the somatosensory cortex of *PV-Syngap1^f/+^* and *Nkx2.1-Syngap1^f/+^* mice.

In the course of the electrophysiological experiments, we made the tentative observation that the number of EGFP^+^ cells appeared decreased in the hippocampus of *Nkx2.1-Syngap1^f/+^* when compared with *Nkx2.1-Syngap1^+/+^* adult mice. In order to determine whether it was indeed the case, we used immunohistochemistry to quantify the number of EGFP^+^PV^+^ and EGFP^+^PV^−^ cells in the hippocampal CA1 and somatosensory cortex from both *Nkx2.1-Syngap1^f/+^* and *Nkx2.1-Syngap1^+/+^* mice. We found that the numbers of EGFP^+^PV^+^ and EGFP^+^PV^−^ cells were significantly decreased in *Nkx2.1-Syngap1^f/+^* mice when compared with their littermate controls (Fig. 8*a*,*b*; Extended Data Fig. 8-1*a*,*b1*,*b2*). Immunohistochemistry performed at P7 also showed a decrease of the number of these cells in the somatosensory cortex of *Nkx2.1-Syngap1^f/+^* mice when compared with *Nkx2.1-Syngap1^+/+^* mice, indicating that their loss occurred early during development (data not shown).

10.1523/ENEURO.0475-22.2023.f8-1 Extended Data Figure 8-1 Number of PV interneurons in the somatosensory cortex of *PV-Syngap1^f/+^* and *Nkx2.1-Syngap1^f/+^* mice. ***a***, Confocal images of coronal sections from the somatosensory cortex (layer 5/6) of *Nkx2.1-Syngap1^+/+^* and *Nkx2.1-Syngap1^f/+^* of adult mice immunostained for GFP (green), PV (magenta), PV/GFP (white), and NeuN (blue). ***b1***, Quantification of the number of PV+GFP+ cells in the somatosensory cortex (layer 5/6) of *Nkx2.1-Syngap1^+/+^* (*n* = 3) and *Nkx2.1-Syngap1^f/+^* (*n* = 6) adult mice. The number of PV+GFP+ were significantly reduced in *Nkx2.1-Syngap1^f/+^* when compared to the *Nkx2.1-Syngap1^+/+^* (unpaired *t* test with Welch’s correction, *p = 0.0052). ***b2***, Quantification of the number of PV-GFP+ cells in the somatosensory cortex (layer 5/6) of *Nkx2.1-Syngap1^+/+^* (*n* = 3) and *Nkx2.1-Syngap1^f/+^* (*n* = 6) adult mice. The number of PV-GFP+ were significantly reduced in *Nkx2.1-Syngap1^f/+^* when compared to the *Nkx2.1-Syngap1^+/+^* mice (Unpaired t test with Welch’s correction, *p = 0.0299). ***c***, Confocal images of coronal sections from the somatosensory cortex (layer 5/6) of *PV-Syngap^+/+^* and *PV-Syngap1^f/+^* of adult mice immunostained for GFP (green), PV (magenta), PV/GFP (white), and NeuN (blue). ***d***, Quantification of the number in the somatosensory cortex (layer 5/6) of PV+ GFP+ cells in *PV-Syngap1^+/+^* (*n* = 3) and *PV-Syngap1^f/+^* (*n* = 3). The number of PV+ GFP+ was not significantly different in *PV-Syngap1^f/+^* when compared to *PV-Syngap1^+/+^* adult mice (unpaired *t* test with Welch’s correction, *p* = 0.2981). Download Figure 8-1, TIF file.

### Inverted *loxP* sites in the *Syngap1^flox^* allele

**Figure 9.**
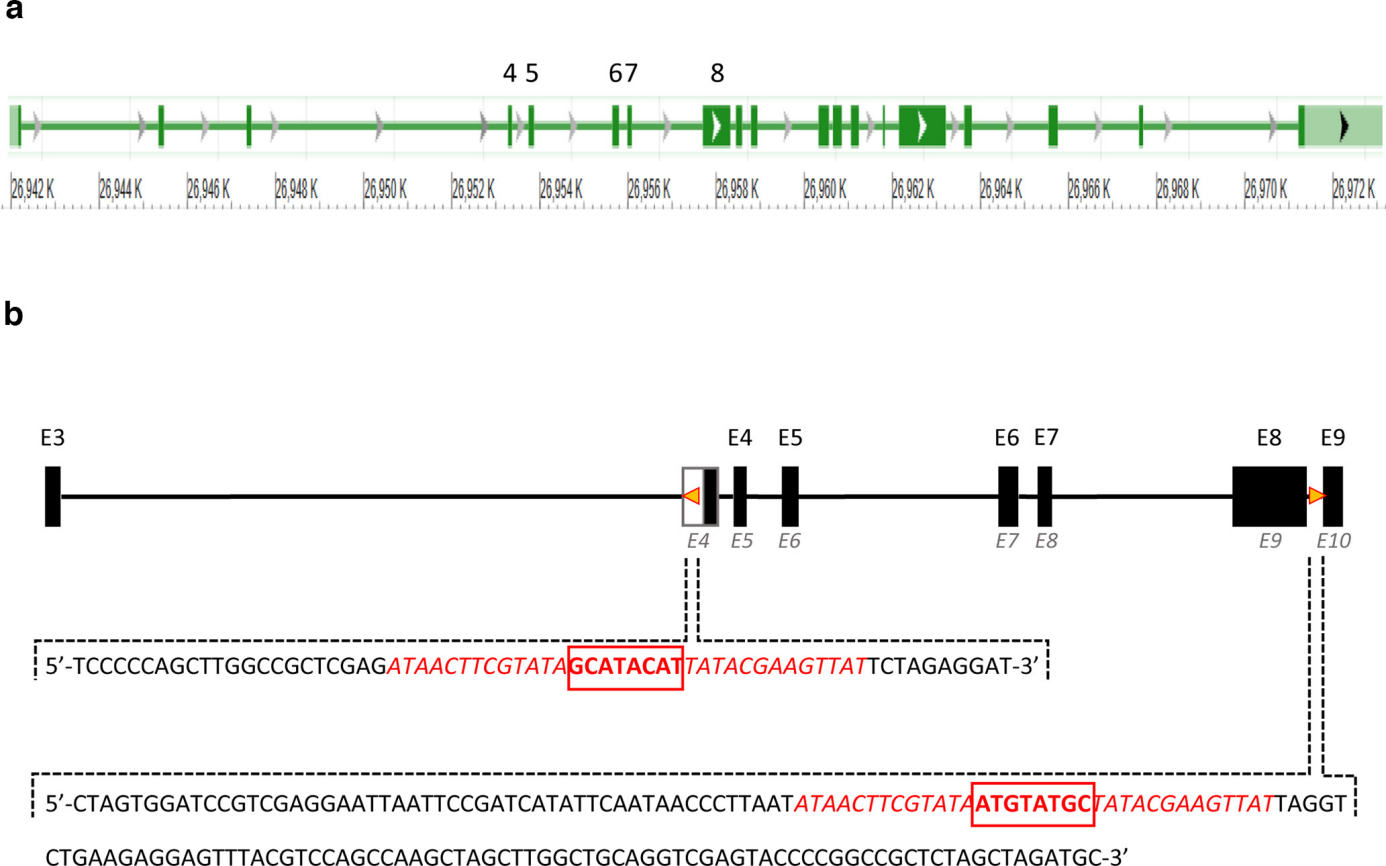
Opposite orientations of loxP sites at the *Syngap1^flox^* allele. ***a***, Genomic organization of the *Syngap1* gene. Shown are the 19 exons (boxes) of RefSeq NC_000083.6 that define the longest mRNA transcript, which codes for the Aα2 protein isoform. LoxP sites are found flanking exons 4–8 (numbered above the diagram). ***b***, *Syngap1* gene segment that harbors the two loxP insertions (yellow triangles) within introns 3 and 8 according to RefSeq (GRCm38). The italicized characters (*E4–E10*) underneath the scheme correspond to the exon numbering used by Vazquez et al. (2004) in their initial description of the *Syngap1^flox^* allele. The half-filled box *E4* represents exon 1 of the transcript coding for the B isoforms with its 5′UTR on the clear side. The sequence of each insertion containing loxP sites (+ strand shown only) is shown as revealed by whole genome sequencing. The intron 3 insertion is 66 bp long whereas the intron 8 insertion is 163 bp. We confirmed that both insertions contain the 34 bp loxP sequence (in red). Each loxP site consists of an 8-bp central spacer region (bold and boxed) that gives the directionality of the site flanked by two 13-bp inverted repeats (italic). Note that each spacer sequence is a reverse complementary with respect to the other.

As *Syngap1* haploinsufficiency in the germline does not affect neuronal cell development or survival in the hippocampus (Knuesel et al., 2005; our unpublished data [Vidya Jadhav (V.J.), Bidisha Chattopadhyaya (B.C.), Maria-Isabel Carreno-Munoz (M.I.C.-M.), Jacques Michaud (J.M.), Graziella Di Cristo (G.D.C.)]), the observation of a decrease of MGE-derived cells in the hippocampus of *Nkx2.1-Syngap1^f/+^* mice was surprising. In order to investigate the mechanism underlying this observation, we sought to sequence the genome of *Syngap1^f/f^* mice to verify the integrity of the *Syngap1^flox^* allele and make sure that *loxP* sites were not found elsewhere in the genome, affecting the expression of other genes than *Syngap1*. Whole-genome sequencing confirmed that *loxP* sites were only found on chromosome 17 within introns 3 and 8 of *Syngap1*, as previously reported (Vazquez et al., 2004). However, the analysis revealed that these two *loxP* sites were in opposite orientations (Fig. 9). Previous studies have established that targeted recombination between inverted *loxP* sites *in cis* can result in the loss of the *loxP*-carrying chromosome in proliferating but not in postmitotic cells because of unequal crossover between sister chromatids after DNA replication and before entry into anaphase (Lewandoski and Martin, 1997; Grégoire and Kmita, 2008). Moreover, these studies have shown that the elimination of the *loxP*-carrying chromosome by targeted recombination may result in cell loss.

**Figure 10.**
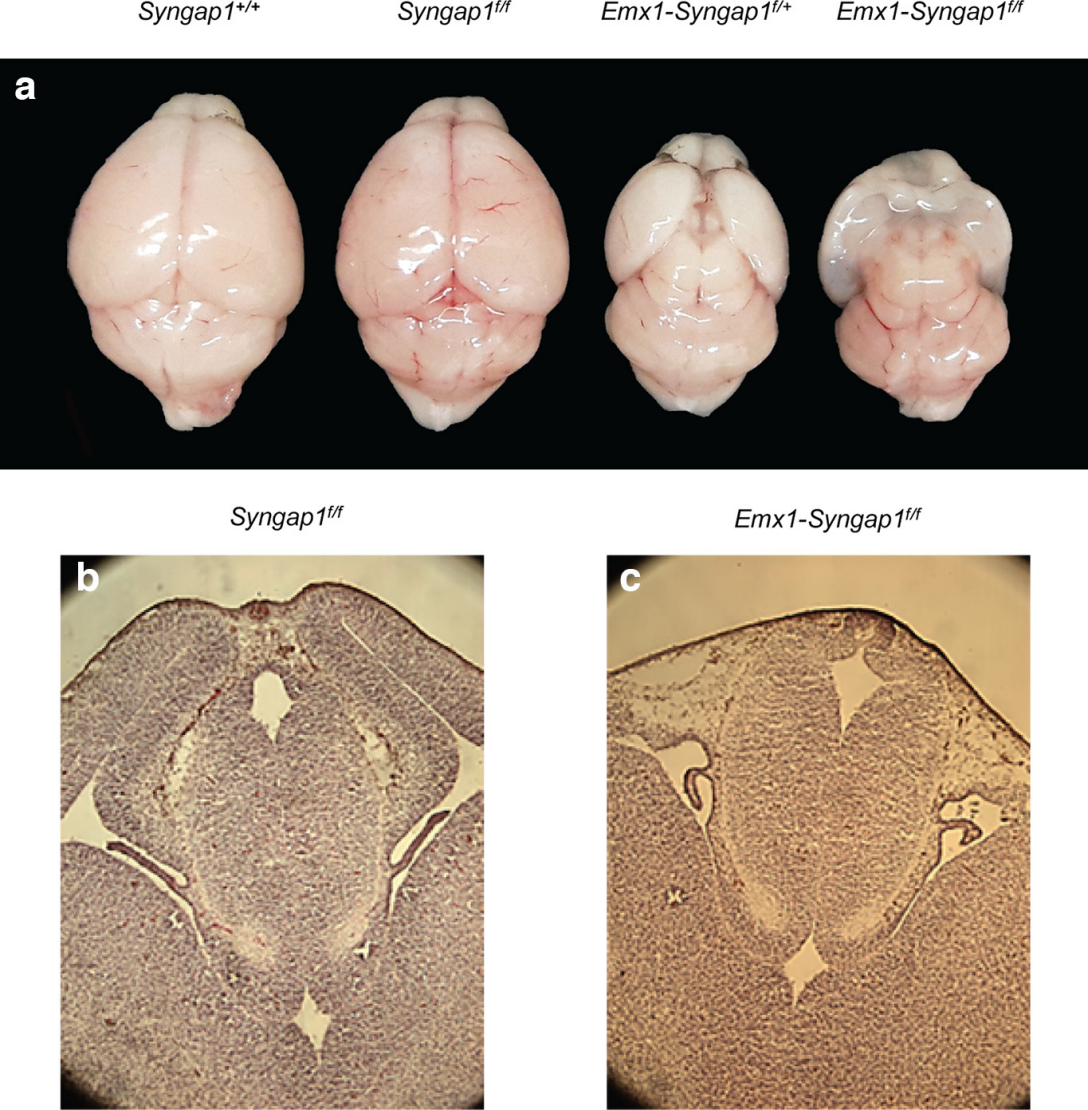
Cortical development in *Emx1-Syngap1^f/+^* and *Emx1-Syngap1^f/f^* mice and embryos. ***a***, Dorsal view of the brain of P24 *Syngap1^+/+^*, *Syngap1^f/+^*, *Emx1-Syngap1^f/+^*, and *Emx1-Syngap1^f/f^* mice. The dorsal telencephalon appears normal in *Syngap1^+/+^* and *Syngap1^f/f^* mice but is underdeveloped in *Emx1-Syngap1^f/+^* and absent in *Emx1-Syngap1^f/f^* mice. b,c. Coronal sections of E13.5 *Syngap1^f/f^* (***b***) and *Emx1-Syngap1^f/f^* (***c***) brains stained with cresyl violet. The cortex is absent in the *Emx1-Syngap1^f/f^* embryo but not in the *Syngap1^f/f^* embryo.

As the *Nkx2.1-cre* transgene is expressed in the ventricular layer of the MGE, one possibility is that recombination between these inverted *loxP* sites leads to the death of proliferating progenitors of MGE-derived hippocampal interneurons, explaining their decrease in *Nkx2.1-Syngap1^f/+^* adult mice (Xu et al., 2008). If this is the case, we would expect that targeted recombination would also induce the loss of other types of proliferating cells in *Syngap1^f/+^* and *Syngap1^f/f^* embryos. In order to investigate this possibility, we induced targeted recombination of the *Syngap1^flox^* allele using the *Emx1-cre* transgene, which is expressed in most excitatory neurons of the cortex and hippocampus and their proliferating progenitors (Gorski et al., 2002). Remarkably, we found that the dorsal telencephalon was severely under-developed in adult *Emx1-Syngap1^f/+^* and *Emx1-Syngap1^f/f^* mice as well as in E13.5 *Emx1-Syngap1^f/f^* embryos when compared with their *Syngap1^+/+^* and *Syngap1^f/f^* counterparts (Fig. 10). This pattern of anomalies is consistent with the loss of proliferating cells on recombination of the *Syngap1^flox^* allele during telencephalon development. In contrast, targeted recombination of another *Syngap1* conditional allele using the *Emx1-cre* transgene was not reported to affect the development of the telencephalon (Ozkan et al., 2014).

The *Nkx2.1-cre* transgene is also expressed postmitotically in the lineage of MGE-derived interneurons populating the hippocampus (Xu et al., 2008). Since targeted recombination between inverted *loxP* induces chromosomal elimination only in proliferating cells, *cre* expression in postmitotic cell should not affect the cellular composition of the hippocampus in *Syngap1^flox/+^* mice. We sought to test this hypothesis by counting the number of MGE-derived cells in *Syngap1^f/+^* mice carrying the *Pv-cre* transgene, which drives the expression of *cre* in postmitotic PV^+^ GABAergic interneurons starting on P12 (Yoon et al., 2000; Moon et al., 2002). We found that the number of EGFP^+^PV^+^ and EGFP^+^PV^−^ cells in the hippocampus and somatosensory cortex of adult *Pv-Syngap1^f/+^* mice was comparable to that of littermate controls (Fig. 8*c*,*d*; Extended Data Fig. 8-1*c*,*d*). Overall, these results are consistent with the possibility that targeted recombination of the *Syngap1^flox^* allele in proliferating but not in postmitotic cells induces cell loss, explaining the reduction of the number of MGE-derived neurons in the hippocampus of *Nkx2.1-Syngap1^f/+^* mice.

**Figure 11.**
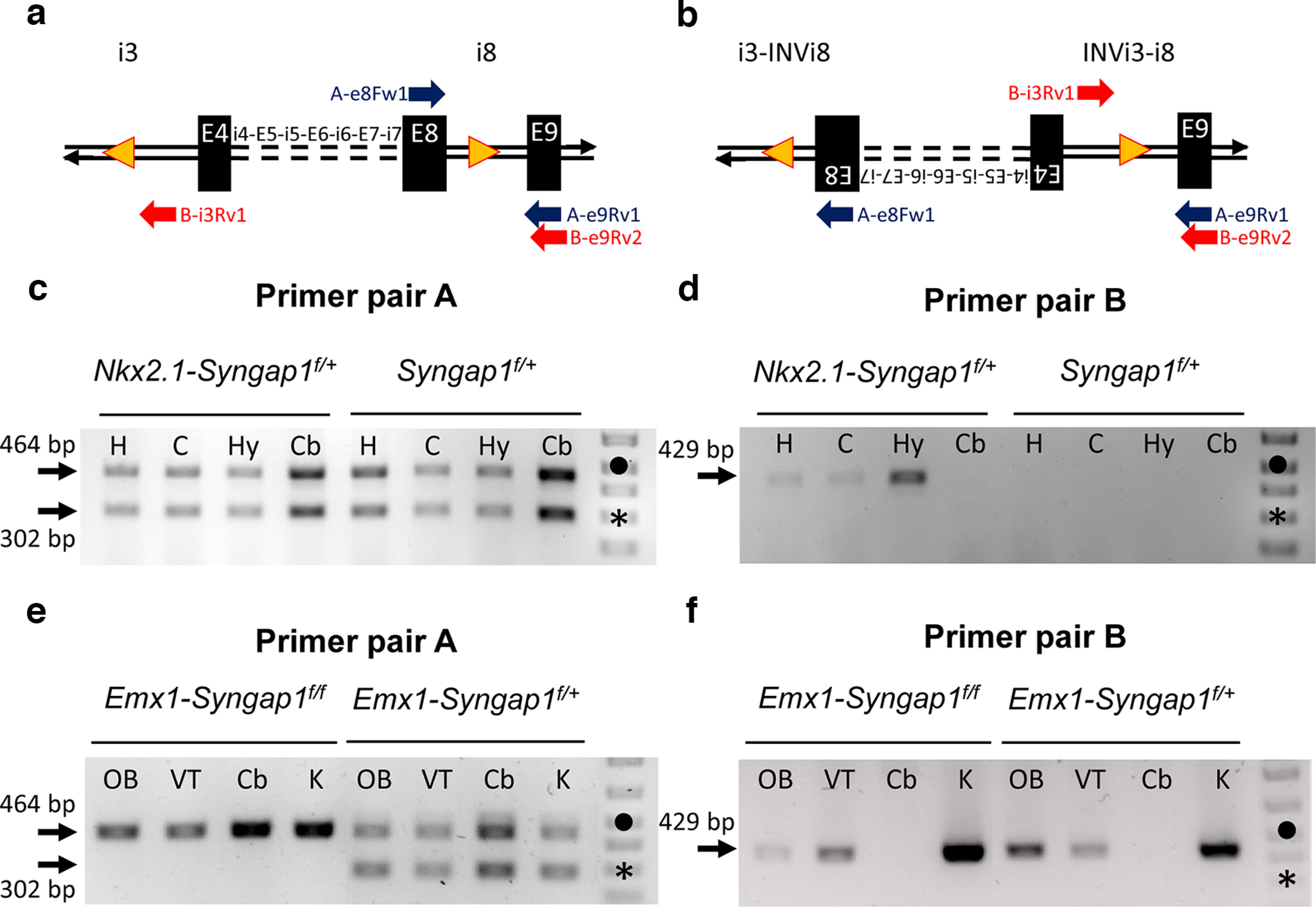
Cre-mediated inversion of *Syngap1*’s floxed sequence. ***a***, ***b***, Schemes (not drawn to scale) showing the relative positions of the *loxP* sites (yellow triangles) and exons 4–8 within the nonrecombined (***a***) and recombined (inverted; ***b***) *Syngap1^flox^* allele. The ∼4.3 kb of intervening introns and exons are depicted by dashed lines. Full horizontal lines with arrowheads represent the double strands of relevant introns. PCR primers belonging to the same pair are illustrated as arrows of the same color with their orientation and approximate annealing location aligned on the corresponding strand. ***c–f***, Agarose gel analyses of PCR products amplified from the region surrounding the loxP site in intron 8 in mice bearing the *Syngap1^flox^* allele with or without transgenes expressing the cre recombinase (*Nkx2.1-* or *Emx1-*). ***c***, ***e***, Amplification using primer pair A (blue) showing products corresponding to the nonrecombined *Syngap1^flox^* allele (464 bp) or to the wild-type allele (302 bp). ***d***, ***f***, Amplification using primer pair B (red) showing the presence or absence of a product predicted to be 429 bp, corresponding to the recombined (inverted) *Syngap1^flox^* allele. Arrows on the left indicate the expected molecular weight of amplicons. Symbols on DNA molecular weight ladder: dot (500 bp) and star (300 bp). Abbreviations: H (hippocampus), C (cerebral cortex), Hy (hypothalamus), Cb (cerebellum), OB (olfactory bulb) VT (ventral telencephalon), K (kidney), bp (base pair).

Postmitotic cre-induced recombination does not induce chromosomal elimination in cells carrying inverted loxP sites, but it does result in the inversion of the sequences flanked by these sites. This inversion event being reversible because of the maintenance of the two *loxP* sites after recombination, ∼50% of cre-expressing postmitotic cells are expected to carry the inverted DNA fragment at any given time point. We sought to determine whether such an inversion event is present in the brain of *Nkx2.1-Syngap1^f/+^* adult mice by performing PCR on genomic DNA (Fig. 11). As expected, we were able to amplify a PCR fragment corresponding to the nonrecombined *Syngap1^flox^* allele from various brain areas of *Nkx2.1-Syngap1^f/+^* and *Syngap1^f/+^* adult mice, including the hippocampus, the cortex, the hypothalamus and the cerebellum (Fig. 11*a*,*c*). In contrast, we were able to amplify a PCR fragment corresponding to the inverted allele only from brain areas of *Nkx2.1-Syngap1^f/+^* mice that express the Tg(*Nkx2.1-Cre*) transgene postnatally or that have been populated by MGE-derived neurons, including the hippocampus, the cortex and the hypothalamus but not the cerebellum (Xu et al., 2008; Fig. 11*b*,*d*). We were not able to amplify this fragment from any brain areas of *Syngap1^f/+^* mice.

We also sought to determine whether the *Syngap1* inversion is present in *Emx1-Syngap1^f/+^* adult mice by performing the same PCR experiments. We were able to amplify the PCR fragments corresponding to the nonrecombined and inverted *Syngap1^flox^* allele from the olfactory bulb, ventral telencephalon, and kidneys of *Emx1-Syngap1^f/+^* and *Emx1-Syngap1^f/f^* mice (Fig. 11*a*,*e*,*f*). In contrast, we were able to detect the nonrecombined but not the inverted allele from the cerebellum of these mice (Fig. 11*a*,*e*,*f*). These results are consistent with the observation that the *Emx1-cre* transgene is expressed in the olfactory bulb, ventral telencephalon, and kidneys but not in the cerebellum (Gorski et al., 2002).

Previous studies have shown that *Nkx2.1* is expressed in all MGE progenitors and many, if not all, MGE postmitotic cells during early development (Sussel et al., 1999; Marin et al., 2000; Nóbrega-Pereira et al., 2008). However, MGE-derived interneurons migrating toward the cortex and hippocampus rapidly downregulate *Nkx2.1* expression after leaving the MGE (Nóbrega-Pereira et al., 2008). The expression of the *Nkx2.1-cre* transgene coincides with that of *Nkx2.1* within the developing MGE but its expression later during development has not been characterized (Xu et al., 2008). Assuming that, like the endogenous gene, the expression of the transgene is downregulated in the MGE-derived interneurons migrating out of the MGE, we would not expect that recombination of the *Syngap1^flox^* allele would occur after the late embryonic stages in these cells.

Recombination of the *Syngap1^flox^* allele in postmitotic cells would result in the inversion of exons 4–8, which encode the entire PH and C2 domains of SYNGAP1. This inversion event is predicted to result in the production of a nonfunctional protein as the C2 domain has been shown to be critical for SYNGAP1 activity (Pena et al., 2008; Meili et al., 2021). Moreover, a previous study has established that targeted recombination of the *Syngap1^flox^* allele in the adult hippocampus reduces SYNGAP1 protein production (Muhia et al., 2012). Interestingly, the reduction of protein levels observed in these experiments is relatively modest, which is consistent with the effect of a cre-induced inversion that would be reversible (see Discussion). All together, these results strongly suggest that the population of hippocampal MGE-derived interneurons of *Nkx2.1-Syngap1^f/+^* mice is divided into two groups, some with two functional copies of *Syngap1* and some with only one copy.

## Discussion

Deleterious mutations in *SYNGAP1* gene cause ID, epilepsy, and ASD in humans (Hamdan et al., 2009, 2011; Berryer et al., 2013). *Syngap1* haploinsufficiency in mice recapitulates several abnormalities observed in human with *SYNGAP1* pathogenic variants, such as deficits in learning and memory and epileptic seizures (Komiyama et al., 2002; Clement et al., 2012; Nakajima et al., 2019). *Syngap1*^+/−^ mice display accelerated maturation of excitatory synapses during sensitive periods of development, leading to an imbalance in excitation-inhibition and an impairment of cognitive processes (Clement et al., 2012, 2013). The role of SYNGAP1 in excitatory neuron network development is well established (Ozkan et al., 2014), but recent reports indicate that it also plays a critical role in the development and maturation of inhibitory synaptic circuits (Berryer et al., 2016; Su et al., 2019). Here, we used a conditional mouse model to uncover the impact of a decrease of *Syngap1* expression in hippocampal inhibitory interneuron function.

### Inverted *loxP* sites in the *Syngap1^flox^* allele

Unexpectedly, we found that the *Syngap1^flox^* allele used in this study contains *loxP* sites that are oriented in opposite directions. The presence of inverted *loxP* sites at the *Syngap1* locus has two potential consequences:

(1) This configuration may induce the elimination of the *Syngap1*-carrying chromosome and subsequent cell loss on recombination between *loxP* sites in proliferating cells. Indeed, we found that *Nkx2.1-cre* expression resulted in a significant decrease of the number of MGE-derived interneurons in the CA1 hippocampus of *Syngap1^f/+^* mice. However, targeted recombination does not affect the development of all MGE-derived interneurons, as a substantial proportion of these cells are generated and populate the hippocampus of *Nkx2.1-Syngap1^f/+^* mice. We hypothesize that MGE progenitors undergo a limited number of cell divisions in the presence of *cre*, allowing a subset of them to escape cell death. We have recently generated a new mouse line using a different *Syngap1* conditional allele (*Syngap1^floxGR^*), where loxP sites flank exons 6–7 of the *Syngap1* gene (Ozkan et al., 2014). We found no significant decrease of cortical and hippocampal MGE-derived interneurons numbers in these Tg(*Nkx2.1-cre*);*Syngap1^floxGR/+^* mice when compared with wild-type littermates, further supporting our conclusion that targeted recombination of the *Syngap1^flox^* allele can induce the death of proliferating cells (unpublished data).

(2) *Cre*-induced recombination between the inverted *loxP* sites in postmitotic cells is predicted to result in the reversible inversion of the *Syngap1^flox^* allele. As expected, we were able to detect both the nonrecombined and the inverted *Syngap1^flox^* allele in brain areas that express the *Nkx2.1-cre* or *Emx1-cre* transgene or that have been populated by cells derived from lineages expressing these transgenes. These results strongly suggest that there are two populations of MGE-derived neurons in the hippocampus of *Nkx2.1-Syngap1^f/+^* adult mice: approximately half of them would carry two functional copies of *Syngap1* while the other half would carry only one functional copy.

Our discovery that the *Syngap1^flox^* allele is composed of inverted *loxP* sites sheds some light on previously published studies involving this mouse line. For instance, Knuesel et al. (2005) reported that the expression of the *Camkii-cre* transgene, which induces targeted recombination in excitatory forebrain neurons beginning approximately one week after birth, did not reduce the level of SYNGAP1 in *Syngap1^ff^* mice below that of the heterozygous KO (Knuesel et al., 2005). Similarly, bilateral injection of a cre-expressing adenoviral vector into the hippocampus of *Syngap1^ff^* mice led only to a modest reduction (24–42%) of SYNGAP1 protein levels (Muhia et al., 2012). It is possible that a greater reduction of SYNGAP1 protein levels was not achieved in these two experiments because the inversion induced by the recombination of the *Syngap1^flox^* allele is reversible in postmitotic cells, resulting in about half of the cells with two functional copies of the *Syngap1* gene.

### Cell type-specific functional alterations in inhibitory interneurons of *Nkx2.1-Syngap1^f/+^* adult mice

A previous study reported that *Nkx2.1-Syngap1^f/+^* mice showed reduced GABAergic neurotransmission, cognitive impairments and social behavioral deficits (Berryer et al., 2016). By performing whole-cell recordings in slice, we found here that MGE-derived interneurons from the hippocampus of these mice display cell-specific impairments. More specifically, we found that Nkx2.1 fast-spiking interneurons (likely corresponding to parvalbumin (PV) expressing interneurons) are characterized by impaired membrane properties and evoked firing (Fig. 1), facilitated AMPAR-mediated, but not NMDAR-mediated, synaptic excitation (Fig. 2) and compromised short-term plasticity of excitatory inputs via a postsynaptic Ca^2+^-dependent mechanism (Fig. 3). In contrast, Nkx2.1 regular-spiking interneurons [likely corresponding to somatostatin (SOM) expressing interneurons] showed intact membrane and firing properties (Fig. 4), synaptic excitation (Fig. 5), and short-term plasticity of excitatory inputs (Fig. 6) comparable to controls. At the network level, the hippocampus of *Nkx2.1-Syngap1^f/+^* mice is characterized by impairment of pyramidal cell synaptic inhibition and enhancement of postsynaptic summation of excitatory responses (Fig. 7).

Overall, our results raise the possibility that conditional decrease of *Syngap1* expression specifically in MGE-derived neurons causes cell type-specific changes that compromise fast-spiking interneuron firing and synaptic function and is associated with impaired pyramidal cell inhibition and enhanced synaptic excitation. Thus, cell-specific alterations in inhibitory interneuron function may contribute to the network hyper-excitability and cognitive impairments in mice models with global *Syngap1* haploinsufficiency (Clement et al., 2012, 2013; Ozkan et al., 2014; Sullivan et al., 2020). Inhibitory interneuron dysfunction may also contribute to seizure susceptibility and cognitive deficits in humans with *SYNGAP1* mutations (Hamdan et al., 2009, 2011; Berryer et al., 2013).

Previous results involving the *Syngap1^flox^* allele (Berryer et al., 2016) and the results reported here have to be interpreted in light of our observation that the *Syngap1^flox^* allele contains inverted loxP sites. On the one hand, it is possible that the decrease of the number of MGE-derived interneurons contributes to the behavioral and cellular deficits of *Nkx2.1-Syngap1^f/+^* mice. For instance, a decrease of these interneurons could contribute to the reduction of the synaptic inhibition of pyramidal cells that we observed in the hippocampus of *Nkx2.1-Syngap1^f/+^* mice. On the other hand, the presence of a nonfunctional allele in only half of the MGE-derived neurons of *Nkx2.1-Syngap1^f/+^* mice could attenuate the effect size of the deficits. However, as outlined below, several of the changes that we documented in MGE-derived interneurons are reminiscent of the role of *Syngap1* in principal excitatory neurons (Clement et al., 2012; Ozkan et al., 2014) or were observed in interneurons in the context of global *Syngap1* haploinsufficiency (Sullivan et al., 2020), suggesting that our observations might be relevant for the understanding of the role of *Syngap1* in inhibitory interneurons.

Our results suggest that hippocampal fast-spiking interneurons from *Nkx2.1-Syngap1^f/+^* mice display intact resting membrane potential but reduced input resistance and impaired firing. The deficit in evoked firing may arise from the changes in input resistance. Interestingly, similar changes in membrane and firing properties were reported in prefrontal cortex PV fast-spiking interneurons in global *Syngap1*^+/−^ mice (Ozkan et al., 2014). Our findings of impaired membrane and firing properties of hippocampal fast-spiking interneurons in *Nkx2.1-Syngap1^f/+^* mice, suggest that the deficits observed in global *Syngap1*^+/−^ mice may result from reduced *Syngap1* expression in inhibitory cells.

At the synaptic level, fast-spiking interneurons from *Nkx2.1-Syngap1^f/+^* mice display a potentiation of AMPAR-mediated synaptic responses with impaired short-term plasticity because of postsynaptic calcium-dependent mechanisms. Our observations of unchanged frequency and increased amplitude of spontaneous EPSCs, increased potency and intact paired-pulse ratio of EPSCs evoked by minimal stimulation, as well as intact NMDAR-mediated EPSCs, suggest that postsynaptic mechanisms such as increased content of AMPARs and/or modification of AMPAR composition mediate synaptic changes in these Nkx2.1^+^ fast-spiking interneurons. Interestingly, potentiation of AMPAR-mediated EPSCs via postsynaptic incorporation of AMPARs takes place in excitatory neurons with global *Syngap1* haploinsufficiency (Clement et al., 2012; Ozkan et al., 2014; Araki et al., 2015). Consistent with our findings, increased expression GluA2 subunits was found in PV interneurons of barrel cortex and CA3 hippocampus of global *Syngap1*^+/−^ mice (Sullivan et al., 2020). Although *Syngap1* regulates activity-induced AMPARs insertion via activation of the Erk and mTOR pathways in excitatory neurons (Wang et al., 2013; Araki et al., 2015), the signaling pathways involved in *Syngap1* regulation of AMPAR function in GABAergic interneurons remain to be determined. Our results of selective impairment of excitatory synaptic transmission in fast-spiking but not regular-spiking MGE-derived interneurons are consistent with the recent report of cell type-specific mechanisms of formation of excitatory synapses on cortical parvalbumin interneurons involving Erb-B2 receptor tyrosine kinase for regulation of the TSC subunit 2 and local control of mRNA translation in parvalbumin but not somatostatin interneurons (Bernard et al., 2022). It will be interesting to examine how SYNGAP1 function may interact with these mechanisms in parvalbumin interneurons.

We observed that short-term facilitation of EPSCs was deficient in hippocampal fast-spiking interneurons from *Nkx2.1-Syngap1^f/+^* mice, suggesting an impairment of temporal integration of synaptic information in these cells. The rescue of short-term facilitation by postsynaptic injection of BAPTA, suggests that the impaired short-term dynamics of excitatory inputs involves Ca^2+^-dependent postsynaptic mechanisms. Deficits in short-term facilitation of synaptic excitation in hippocampal fast-spiking interneurons contribute to network dysfunction in mouse models of ASD and Alzheimer’s disease (Polepalli et al., 2017; Park et al., 2020). In these studies, the deficit of short-term facilitation is mediated by a presynaptic increase in release probability (Polepalli et al., 2017; Park et al., 2020). More generally, short-term synaptic plasticity is mediated by presynaptic and postsynaptic mechanisms such as modification of release probability (Fischer et al., 1997; Zucker, 1999) and modulation of AMPAR dynamics in the postsynaptic membrane (Jones and Westbrook, 1996; Heine et al., 2008; Choquet and Triller, 2013; Constals et al., 2015). Interestingly in hippocampal pyramidal neurons, activity-dependent increase of postsynaptic Ca^2+^ promotes depression of AMPAR-mediated EPSC (Heine et al., 2008). However, whether similar mechanisms occur in inhibitory neurons with *Syngap1* haploinsufficiency remains unknown. Moreover, activity-dependent postsynaptic Ca^2+^ levels determine the direction of long-term plasticity at excitatory synapses of CA1 fast-spiking parvalbumin interneurons, with weak and strong postsynaptic Ca^2+^ increases inducing long-term potentiation and depression respectively (Camiré and Topolnik, 2014). In our experiments, postsynaptic BAPTA injection reversed the loss of short-term potentiation in Nkx2.1^+^ fast-spiking interneurons from *Nkx2.1-Syngap1^f/+^* mice, indicating the involvement of a postsynaptic Ca^2+^ mechanism. Since AMPAR-mediated synaptic responses are increased in Nkx2.1^+^ fast-spiking interneurons from *Nkx2.1-Syngap1^f/+^* mice, it is plausible that larger increases in postsynaptic Ca^2+^ are generated during repetitive stimulation. Such a larger postsynaptic Ca^2+^ rise could elicit short-term depression, rather than the usual short-term facilitation observed in wild-type mice. However, whether such a mechanism occurs remains to be determined.

Since fast-spiking Nkx2.1^+^ interneurons in/near stratum pyramidale and regular-spiking Nkx2.1^+^ interneurons in stratum oriens near the alveus likely correspond to parvalbumin and somatostatin interneurons respectively, our results suggest cell-specific roles of SYNGAP1 in these inhibitory interneurons. Whether these cell-specific roles are because of different expression level of *Syngap1*, or expression of different isoforms of SYNGAP1, or different sensitivity to *Syngap1* dosage (McMahon et al., 2012; Araki et al., 2020; Gou et al., 2020) remains to be determined.

Our findings suggest that changes in intrinsic and firing properties of Nkx2.1 fast-spiking interneurons with *Syngap1* disruption may also contribute to the deficit in synaptic inhibition by these cells. Thus, a firing impairment of Nkx2.1 fast-spiking interneurons likely also contributes to the deficiency in interneuron function by reducing their output function. Collectively, our findings of impaired intrinsic, firing and synaptic properties of Nkx2.1 fast-spiking interneurons were associated with a facilitation of summation of excitatory synaptic responses in hippocampal pyramidal neurons, indicating that a decrease of *Syngap1* in inhibitory interneurons may be sufficient to contribute to network hyperexcitability associated with global *Syngap1* haploinsufficiency (Clement et al., 2012; Ozkan et al., 2014).

Previous studies reported a decrease of perisomatic PV^+^ punctae in specific areas of the cortex in *Syngap1*^+/−^ mice (Berryer et al., 2016; Sullivan et al., 2020). Moreover, biolistic expression of cre in PV^+^ cells from postnatal *Syngap1^f/f^* cortical organotypic slices reduces the formation of perisomatic boutons (Berryer et al., 2016). Overall, these observations suggest that SYNGAP1 promotes the formation of PV^+^ cell innervations in a cell-autonomous manner. Interestingly, a similar decrease of perisomatic PV^+^ punctae was found in the cortex of *Nkx2.1-Syngap1^f/+^* mice, raising the possibility that the synaptic changes in MGE-derived interneurons reported here contribute to the innervation deficits associated with the disruption of *Syngap1* (Berryer et al., 2016).

In conclusion, although our results suggest that *Syngap1* plays a specific role in inhibitory neurons for the development of neuronal circuits involved in cognition and behavior, it will be important to study the function of these cells using a different *Syngap1* conditional allele in light of our discovery that the *Syngap1^flox^* allele used in this study carries inverted *loxP* sites. Interestingly, we recently found that Tg(*Nkx2.1-cre*);*Syngap1^floxGR/+^* mice show cognitive alterations, including social behavioral deficits, supporting the hypothesis that *Syngap1* plays a role in the establishment of GABAergic circuit function (unpublished data; Berryer et al., 2016; Su et al., 2019). This mouse line would thus be suitable for further studying the role of *Syngap1* in inhibitory interneurons using whole-cell recordings.

## Synthesis

Reviewing Editor: Jean Christophe Poncer, INSERM Decisions are customarily a result of the Reviewing Editor and the peer reviewers coming together and discussing their recommendations until a consensus is reached. When revisions are invited, a fact-based synthesis statement explaining their decision and outlining what is needed to prepare a revision will be listed below. The following reviewer(s) agreed to reveal their identity: Corentin Le Magueresse, Peter Blaesse.

Both reviewers acknowledge that your observations, despite the unexpected observation that Syngap1flox mice have inverted loxP sites, will be useful to the community and that most of the experiments were carefully performed.

However, they note that the manuscript in its current form suffers from a lack of rigor in the statistical analysis of the data, and a lack of power in some of the statistics due to the small number of observations. We urge that the number of observations for the corresponding data be increased to achieve acceptable statistical power, and that appropriate statistical tests be employed (as recommended by reviewer 1). Although it would be interesting to compare the neural properties of PV-cre/Syngap1flox mice, we understand that you may not be ready to commit to this additional set of experiments and we leave it to you to follow the reviewer’s suggestion in this regard.

---

Reviewer 1

This study investigates the role of Syngap1 disruption on the function of two types of MGE-derived hippocampal CA1 interneurons, namely Fast-Spiking (FS) and Regular-Spiking (RS) cells.

SYNGAP1 haploinsufficiency is a well-established cause of intellectual disability. Over the past 20 years, mouse models with Syngap1 haploinsufficiency have been used to study the role of Syngap1 in neuronal maturation and function. In particular, two distinct floxed Syngap1 mouses line have been generated to study the cell type-specific and brain region-specific consequences of Syngap1 haploinsufficiency. Previously, one of these lines was crossed with Nkx2.1-Cre mice to generate Nkx2.1-Cre;Syngap1flox/+mice and used to demonstrate that Syngap1 haploinsufficiency in MGE-derived interneurons leads to deficits in the GABAergic innervation of cortical pyramidal cells (Berryer et al., 2016). However, the consequences of Syngap1 haploinsufficiency on the innervation and functional properties of GABAergic interneurons remain unknown.

Here, the authors cross another, distinct floxed Syngap1 mouse line with Nkx2.1-Cre mice and RCE reporter mice to generate triple transgenic Nkx2.1-Cre;RCE;Syngap1flox/+mice. They report that FS and RS interneurons in mutant mice display different synaptic and excitability deficits. Crucially, they also observe that the mutation results in cell loss, which is at odds with published results using the other floxed Syngap1 mouse line. Using whole-genome sequencing they find that the floxed Syngap1 allele contains inverted loxP sites, indicating that mutant mice do not exhibit haploinsufficiency of Syngap1 in MGE-derived neurons, but rather an unquantified disruption of Syngap1 expression.

Overall, the experiments presented throughout the manuscript appear to be technically sound, albeit with insufficient statistical power in several cases. The authors are to be commended for carefully analyzing the reasons for the discrepancies between their results and the results based on another floxed mouse line. The choice of the floxed Syngap1 with an inverted loxP site is unfortunate, as the results presented in the manuscript do not reflect Syngap1 haploinsufficiency. Nevertheless, they reflect disruption of Syngap1 function in postmitotic cells, and the differences between FS cells and RS cells in mutant mice are of interest. Furthermore, it is important that the presence of the inverted loxP site in the floxed Syngap1 mouse line be brought to the attention of the scientific community - this would constitute a useful contribution to the self-correcting process of science.

Some conclusions, however, are insufficiently backed by the data.

Specific points:

1) In general, when multiple comparisons are performed using the Mann-Whitney test (because 2 way ANOVAs cannot be used), Bonferroni corrections should be applied. In general, the purpose of using a Friedmann ANOVA and what the result indicates should be more clearly stated.

2) Intrinsic excitability (Fig.1d and Fig.4d). The frequency-current intensity curves should be shown in their entirety, together with the appropriate statistical analysis (repeated measure 2-way ANOVA when possible; or with multiple Mann-Whitney tests, with Bonferroni corrections). In particular, the rheobase appears to be nearly twice higher in RS cells from mutant mice (Fig 4f), but the number of recordings is too low for this experiment to be conclusive. It is necessary to increase the number of recordings in this condition. Moreover, it is unclear whether those frequency-intensity measurements were performed in the presence of blockers of synaptic transmission. Please specify.

3) NMDA receptor -mediated currents (Fig.2 l-n). The claim that NMDAR-mediated EPSCs are unchanged in FS cells, unlike AMPAR-mediated currents, is not properly substantiated. In fact, NMDAR-mediated currents do appear to be increased, but the small number of recordings and the high variability in the mutant condition make a proper assessment difficult. The number of recordings should be increased in this condition. Furthermore, the authors should measure the AMPA/NMDA ratio, for instance by measuring the peak of the AMPAR-mediated current at hyperpolarizing potentials, and measuring the NMDAR-mediated current at depolarized potentials 50ms after the stimulation artefact. Only then will it be possible to conclude that there is a difference between AMPAR- and NMDAR-mediated currents.

In fig. 2m specifically: the * seems to indicate a difference in the Mann-Whitney test, but this seems at odds with the data presented on the graph. Can the authors check?

4) The authors claim that sEPSC amplitudes are similar in RS cells from control and mutant mice (Fig5 a-e). In contrast, evoked PSC amplitudes significantly differ in the two conditions. This discrepancy is not discussed. Since there is a tendency towards decreased sEPSC amplitude in mutant mice and a low number of recordings, more recordings should be carried out. If discrepancies between sEPSC and eEPCS amlitudes persist, hypotheses should be proposed to explain them.

5) Conclusions based on the results presented in 6 (short-term facilitation) should be phrased more carefully (lines 423-432). The Friedman ANOVA does not allow to test the differences between wild-types and mutants, and short-term plasticity in mutant mice might be slightly changed, according to the graphs. Do small p values (Friedman ANOVA p < 0.05) indicate that there is some degree of short-term plasticity at the synapse? This should be explained more clearly.

Symbols showing the results of the ANOVA in Fig. 3 and 6 should differ from symbols showing the results of the Mann-Whitney tests.

6) The last sentence of the results part (“these results strongly suggest that the population of [...] interneurons [...] is divided into two groups, some with 2 functional copies of Syngap1 and some with only one copy” lines 556-559) needs further elaboration. Similar comments apply to lines 600-603. The authors correctly argue that inversion yields two loxP sites that are indistinguishable from the original loxP pair due to the conservative nature of the recombination event. Therefore, recombination must continue as long as Cre is present. How long is Cre expressed in post-mitotic Nkx2.1+ neurons in Nkx2.1-Cre mice ? If Cre remains expressed until adulthood, then one would expect that the continuous inversion of one Syngap1 allele leads to waves of Syngap1 expression from the floxed allele, interleaved with periods of reduced expression (when the floxed gene is inverted), in all MGE-derived neurons. If, on the other hand, Cre expression stops at some point during development (if so, when?), then the floxed allele of Syngap1 will take a definitive position (either normal or inverted), and two distinct groups of neurons will be generated - some lacking one copy of Syngap1. It is important to stress that even in the latter case, the developmental trajectory of these two neuronal groups will be indistinguishable as long as Cre is expressed.

7) In the discussion the authors could elaborate briefly on the differences between FS and RS cells in view of the recently published paper by the group of O. Marin, which shows that the mechanisms governing synapse formation in PV+ and SOM+ interneurons differ (doi: 10.1126/science.abm7466).

Typos:

Line 78: mouse models

Line 88: of the GluA2 subunit

Line 91: although these lines

Line 331: in the Input-Output (I-O) relationship

---

Reviewer 2

In the presented manuscript, the authors describe the effect of a conditional knockout of Syngap1 (Syngap1flox mouse line by Vasquez et al., 2004) in Medial Ganglionic Eminence (MGE)-derived hippocampal interneurons (Nkx2.1-Cre promoter line) on the electrophysiological properties of those interneurons and on network properties. In addition, triggered by the surprising finding of a reduced number of hippocampal interneurons, the authors were able to use whole genome sequencing to show that the loxP sites in Syngap1flox mice are inverted.

Obviously, the knowledge gained in the course of the study about the incorrect orientation of the loxP sites and the resulting consequences are an obstacle to a clear interpretation of the electrophysiological effects. Since the finding of the inverted loxP sites is of great relevance for already published papers and since the authors meaningfully discuss their own electrophysiological findings with regard to the inverted loxP sites, the manuscript represents an important contribution to the field despite this limitation.

However, a number of concerns should be addressed before publication:

1. In an overall balanced discussion, the authors emphasize cell-type-specific differences in Syngap1 disruption between fast-spiking and regular-spiking neurons (FS-INs and RS-INs). This difference is not entirely clear from the data. The effects on input resistance and rheobase are not fundamentally different and the significance level is only just missed for the RS-INs (with lower n-numbers). Especially since an unknown number of the recorded cells shows normal SYNGAP1 levels due to the inverted loxP sites, it is not clear whether the lack of an effect in RS-INs is sufficiently proven.

2. It is not clear whether the statistical analysis (Mann-Whitney test) used for data shown in Figs. 2f,m, 3 and 5d is appropriate (cf. Fig. 7e).

3. The authors used PV-cre/Syngap1flox mice for the comparison of cell loss. It would be interesting to check FS-IN properties and excitatory responses in pyramidal cells in those mice.

4. The authors should define meaningful abbreviations for the mouse strains used and use them consistently throughout the manuscript. Using “Tg(Nkx2.1-Cre);RCEflox/flox;Syngap1flox/+ and Tg(Nkx2.1-Cre);RCEflox/flox;Syngap1+/+” is not reader-friendly and switching between “CTRL”, “WT”, cHET” and other nomenclature is misleading.

5. Explain numbers given in summary graphs (e.g., “13 (6)”).

6. Figs. 3c,d: Is there direct statistical evidence for the rescue of short-term facilitation by BAPTA in cHET cells?

Minor:

Define the criteria for “stable” holding currents and series resistance.

l. 224: define temperature

l. 227: No wash steps between primary and secondary antibody incubation?

l. 231: Provide correct information for the anti-chicken antibody used.

l. 240: Explain “PV-GFPhg”.

l. 414: INs

l. 1073: “c-f”

l. 1087: define Cre-promoter line

“EGFP” vs. “GFP”

“flox” vs. “flx”

## Author Response

Synthesis of Reviews:

Synthesis Statement for Author (Required):

Both reviewers acknowledge that your observations, despite the unexpected observation that Syngap1flox mice have inverted loxP sites, will be useful to the community and that most of the experiments were carefully performed.

However, they note that the manuscript in its current form suffers from a lack of rigor in the statistical analysis of the data, and a lack of power in some of the statistics due to the small number of observations. We urge that the number of observations for the corresponding data be increased to achieve acceptable statistical power, and that appropriate statistical tests be employed (as recommended in the review).

As suggested, we addressed the concerns about the statistical analysis and the number of observations, as described in detail below (Reviewer 1, points 1-5; Reviewer 2, points 1-2)

Although it is interesting to compare the neural properties of PV-cre/Syngap1flox mice, we understand that you may not be ready to commit to this additional set of experiments and we leave it to you to follow the reviewer’s suggestion in this regard.

As detailed below (Reviewer 2 point 3), we agree that comparing the neural properties of PV-cre/Syngap1flox mice would be interesting, but we considered these additional experiments beyond the scope of the present paper.

---

REVIEWER 1

This study investigates the role of Syngap1 disruption on the function of two types of MGE-derived hippocampal CA1 interneurons, namely Fast-Spiking (FS) and Regular-Spiking (RS) cells. SYNGAP1 haploinsufficiency is a well-established cause of intellectual disability. Over the past 20 years, mouse models with Syngap1 haploinsufficiency have been used to study the role of Syngap1 in neuronal maturation and function. In particular, two distinct floxed Syngap1 lines have been generated to study the cell type-specific and brain region-specific consequences of

Syngap1 haploinsufficiency. Previously, one of these lines was crossed with Nkx2.1-Cre mice to generate Nkx2.1-Cre;Syngap1flox/+ mice and used to demonstrate that Syngap1 haploinsufficiency in MGE-derived interneurons leads to deficits in the GABAergic innervation of cortical pyramidal cells (Berryer et al., 2016). However, the consequences of Syngap1 haploinsufficiency on the innervation and functional properties of GABAergic interneurons remain unknown.

Here, the authors cross another, distinct floxed Syngap1 mouse line with Nkx2.1-Cre mice and 2 RCE reporter mice to generate triple transgenic Nkx2.1-Cre;RCE;Syngap1flox/+mice. They report that FS and RS interneurons in mutant mice display different synaptic and excitability deficits.

Crucially, they also observe that the mutation results in cell loss, which is at odds with published results using the other floxed Syngap1 mouse line. Using whole-genome sequencing they find that the floxed Syngap1 allele contains inverted loxP sites, indicating that mutant mice do not exhibit haploinsufficiency of Syngap1 in MGE-derived neurons, but rather an unquantified disruption of Syngap1 expression.

Overall, the experiments presented throughout the manuscript appear to be technically sound, albeit with insufficient statistical power in several cases. The authors are to be commended for carefully analyzing the reasons for the discrepancies between their results and the results based on another floxed mouse line. The choice of the floxed Syngap1 with an inverted loxP site is unfortunate, as the results presented in the manuscript do not reflect Syngap1 haploinsufficiency. Nevertheless, they reflect disruption of Syngap1 function in postmitotic cells, and the differences between FS cells and RS cells in mutant mice are of interest. Furthermore, it is important that the presence of the inverted loxP site in the floxed Syngap1 mouse line be brought to the attention of the scientific community - this would constitute a useful contribution to the self-correcting process of science.

We thank the reviewer for considering our results of interest and a useful scientific contribution.

Some conclusions, however, are insufficiently backed by the data.

Specific points:

1) In general, when multiple comparisons are performed using the Mann-Whitney test (because

2 way ANOVAs cannot be used), Bonferroni corrections should be applied. In general, the purpose of using a Friedmann ANOVA and what the result indicates should be more clearly stated.

As suggested by the Reviewer, we revised our statistical analyses of the data for multiple comparisons of repeated measures. In all cases, we tested for data normality and homoscedasticity (Shapiro-Wilk and Levene tests respectively). If data data passed normality and homoscedasticity assumptions, we used a mixed repeated-measure ANOVA to test for significant difference between genotypes (ex. Figure 1e). We did not perform multiple

Bonferroni pair-wise comparisons, but measured the linear regression of repeated measures to obtain the gain (slope) of input-output functions (cell firing or synaptic current) and compared between genotypes (ex. Figure 1e and f). If normalcy test failed, we used one-way

Friedmann ANOVA to determine significance of repeated-measures for each genotype (time effect; ex Figure 3a) and followed by a Mann-Whitney test for a single pair-wise comparisons of the last data pair (end of time series) to test for significant difference between genotypes (ex. Figure 3a). The text was modified accordingly in the Methods (lines 212-214, 292), in the statistical table (Table1-1), and in Figure legends 1-7. 3

2) Intrinsic excitability (Fig.1d and Fig.4d). The frequency-current intensity curves should be shown in their entirety, together with the appropriate statistical analysis (repeated measure 2- way ANOVA when possible; or with multiple Mann-Whitney tests, with Bonferroni corrections).

As suggested by the reviewer, we added plots of frequency-current intensity curves in their entirety in Figures 1e and 4e. Mixed repeated-measures ANOVAs were used to determine significant difference between genotypes. In addition, F/I regression lines are shown in Figures 1e and 4e to illustrate F/I gain, with genotype differences illustrated in Figure 1f and 4f.

Results section (lines 321; 404), Figures 1 and 4 and their legends, and statistical table 1-1, were modified accordingly.

In particular, the rheobase appears to be nearly twice higher in RS cells from mutant mice (Fig 4f), but the number of recordings is too low for this experiment to be conclusive. It is necessary to increase the number of recordings in this condition. Moreover, it is unclear whether those frequency-intensity measurements were performed in the presence of blockers of synaptic transmission. Please specify.

In the experiments with RS cell of Figure 4g (previous 4f), the number of cells is 10 wt vs 7 tg, which are of the same order of magnitude as in experiments with FS cells of Figure 1g (13 wt and 8 tg) for which the difference was significant. We performed a power analysis of the data of Figure 4g and determined that the sample size of 48 cells would be necessary to reach significance, which is biologically unrealistic and thus support our conclusion of no differences in RS cells. Results of the power analysis were added in Methods (line 295-296), Figure 4g legend, and the statistical table 1-1.

As requested by the reviewer, we also clarified in the Methods (lines 158-159) that the F-I measurements were performed in the absence of blockers of synaptic transmission.

3) NMDA receptor -mediated currents (Fig.2 l-n). The claim that NMDAR-mediated EPSCs are unchanged in FS cells, unlike AMPAR-mediated currents, is not properly substantiated. In fact, NMDAR-mediated currents do appear to be increased, but the small number of recordings and the high variability in the mutant condition make a proper assessment difficult. The number of recordings should be increased in this condition.

As suggested by the reviewer point 1, we re-analyzed the data using a mixed repeated measure ANOVA to determine significance of genotype effect for the data in Fig 2f and 2m. These results indicate significant differences between genotypes in AMPAR- (Figure 2f) but not NMDAR-mediated (Figure 2m) EPSCs. In addition, the I/O slope was significantly different between genotypes for AMPAR- (Figure 2f) but not NMDAR-mediated (Figure 2m) EPSCs. We performed a power analysis of the data of Fig 2n and determined that a sample size of 54 cells would be necessary to reach significance, which is biologically unrealistic and thus support our conclusion of no differences in NMDAR-mediated EPSCs. Results (lines 337-339), Figure 2f and m and its legend, and the statistical table 1-1 were modified accordingly. 4

Furthermore, the authors should measure the AMPA/NMDA ratio, for instance by measuring the peak of the AMPAR-mediated current at hyperpolarizing potentials, and measuring the NMDAR-mediated current at depolarized potentials 50ms after the stimulation artefact. Only then will it be possible to conclude that there is a difference between AMPAR- and NMDAR-mediated currents.

We respectfully disagree with the reviewer on this point. The direct measurement of the input-output function of AMPAR-EPSC and/or NMDAR-EPSC in pharmacological isolation is the best experimental strategy to determine if each synaptic current is altered, especially when one is affected but not the other (as in our experiments). The AMPA/NMDA ratio would be subject to change if either (or even both) AMPAR EPSC or NMDAR EPSC would be altered. Thus, we feel that although these complementary experiments would be interesting, they are beyond the scope of the present experiments.

In fig. 2m specifically: the * seems to indicate a difference in the Mann-Whitney test, but this seems at odds with the data presented on the graph. Can the authors check? After the revised statistical analysis indicated in point 1 and above, there is no significant difference. These results were modified in Fig 2m and legend.

4) The authors claim that sEPSC amplitudes are similar in RS cells from control and mutant mice (Fig5 a-e). In contrast, evoked PSC amplitudes significantly differ in the two conditions. This discrepancy is not discussed. Since there is a tendency towards decreased sEPSC amplitude in mutant mice and a low number of recordings, more recordings should be carried out. If discrepancies between sEPSC and eEPCS amlitudes persist, hypotheses should be proposed to explain them.

After the revised statistical analysis (indicated in point 1) for the data in Figure 5d, the mixed repeated measures ANOVA showed no differences in evoked EPSCs between genotypes. In addition, this is consistent with the I/O slope of evoked EPSCs being not different between genotypes. We added the linear fit of the I/O function in Figure 5d, and modified accordingly the results in the text (lines 212-214, 415-417, 636), Figure 5d and its legend, and statistical table 1-1. Regarding the number of recordings in these experiments, we performed a power analysis of the data of Fig 5b and determined that a sample size of 106 cells would be necessary to reach significance for sEPSC frequency. We performed a power analysis of the data of Fig 5c and determined that a sample size of 118 cells would be necessary to reach significance for sEPSC amplitude. We performed a power analysis of the data of Fig 5e and determined that a sample size of 104 cells would be necessary to reach significance for EPSC I/O slope. These required sample sizes are biologically unrealistic and thus support our conclusion of no differences in spontaneous and evoked EPSCs between genotypes. The results of the power analyses were added in the legend of Figure 5 and in the statistical table 1-1. 5

5) Conclusions based on the results presented in 6 (short-term facilitation) should be phrased more carefully (lines 423-432). The Friedman ANOVA does not allow to test the differences between wild-types and mutants, and short-term plasticity in mutant mice might be slightly changed, according to the graphs. Do small p values (Friedman ANOVA p < 0.05) indicate that there is some degree of short-term plasticity at the synapse? This should be explained more clearly.

As requested by the reviewer (and as indicated in point 1 above with the revised statistical analysis), we clarified and phrased more carefully the conclusions of the results of short-term facilitation in Figures 3 and 6. We clarified the results testing for short-term plasticity in each genotype (using Friedman ANOVA), and testing for a difference between genotypes with a single pairwise comparison of EPSC amplitude ratio for stimulus #10 at the end of the train (using a Mann-Whitney test). Our results indicate that short-term facilitation in FS cells is impaired in Nkx2.1-Syngap1f/+ mice in Figure 3, and that short-term facilitation in RS cells is mostly unchanged in Nkx2.1-Syngap1f/+ mice in Figure 6. We modified accordingly the text in the Results (lines 366-369; 427-430), Figures 3 and 6 and their respective legends, and the statistical table 1-1.

Note that for the BAPTA experiments in Fig. 3d, although the data distribution was normal, we used the non-parametric statistical analyses (Friedman ANOVAs and Mann-Whitney test) for comparing results with those of Fig. 3c (and indicating rescue by BAPTA).

Using parametric statistical analysis yielded the same results (significant facilitation and no difference between genotypes).

Symbols showing the results of the ANOVA in Fig. 3 and 6 should differ from symbols showing the results of the Mann-Whitney tests.

As suggested by the reviewer, we used different symbols to indicate significance for the Mann-Whitney pair-wise comparison (#; and color shading) and for the Friedman ANOVA (*) in Figs 3 and 6. Figures and legends were modified accordingly.

6) The last sentence of the results part (“these results strongly suggest that the population of [...] interneurons [...] is divided into two groups, some with 2 functional copies of Syngap1 and some with only one copy” lines 556-559) needs further elaboration. Similar comments apply to lines 600-603. The authors correctly argue that inversion yields two loxP sites that are indistinguishable from the original loxP pair due to the conservative nature of the recombination event. Therefore, recombination must continue as long as Cre is present. How long is Cre expressed in post-mitotic Nkx2.1+ neurons in Nkx2.1-Cre mice ? If Cre remains expressed until adulthood, then one would expect that the continuous inversion of one Syngap1 allele leads to waves of Syngap1 expression from the floxed allele, interleaved with periods of reduced expression (when the floxed gene is inverted), in all MGE-derived neurons. If, on the other hand, Cre expression stops at some point during development (if so, when?), then the floxed allele of

Syngap1 will take a definitive position (either normal or inverted), and two distinct groups of neurons will be generated - some lacking one copy of Syngap1. It is important to stress that 6 even in the latter case, the developmental trajectory of these two neuronal groups will be indistinguishable as long as Cre is expressed.

As requested by the reviewer, we elaborated on the findings at the end of results (lines 545- 556).

7) In the discussion the authors could elaborate briefly on the differences between FS and RS cells in view of the recently published paper by the group of O. Marin, which shows that the mechanisms governing synapse formation in PV+ and SOM+ interneurons differ (doi: 10.1126/science.abm7466).

As suggested by the reviewer, we elaborated on our results in view of the recent paper of Oscar Marin group in the discussion (lines 688-695) and added the reference in the list (Line 778).

Typos:

Line 79 (previous L78): mouse models. Corrected

Line 90 (previous L88): of the GluA2 subunit. Corrected

Line 93 (previous L 91): although these lines. Corrected

Line 337 (previous L331): in the Input-Output (I-O) relationship. Corrected

----

REVIEWER 2

In the presented manuscript, the authors describe the effect of a conditional knockout of Syngap1 (Syngap1flox mouse line by Vasquez et al., 2004) in Medial Ganglionic Eminence (MGE)- derived hippocampal interneurons (Nkx2.1-Cre promoter line) on the electrophysiological properties of those interneurons and on network properties. In addition, triggered by the surprising finding of a reduced number of hippocampal interneurons, the authors were able to use whole genome sequencing to show that the loxP sites in Syngap1flox mice are inverted.

Obviously, the knowledge gained in the course of the study about the incorrect orientation of the loxP sites and the resulting consequences are an obstacle to a clear interpretation of the electrophysiological effects. Since the finding of the inverted loxP sites is of great relevance for already published papers and since the authors meaningfully discuss their own electrophysiological findings with regard to the inverted loxP sites, the manuscript represents an important contribution to the field despite this limitation.

We thank the reviewer for considering our manuscript an important contribution to the field.

However, a number of concerns should be addressed before publication:

1. In an overall balanced discussion, the authors emphasize cell-type-specific differences in

Syngap1 disruption between fast-spiking and regular-spiking neurons (FS-INs and RS-INs). This difference is not entirely clear from the data. The effects on input resistance and rheobase are 7 not fundamentally different and the significance level is only just missed for the RS-INs (with lower n-numbers). Especially since an unknown number of the recorded cells shows normal SYNGAP1 levels due to the inverted loxP sites, it is not clear whether the lack of an effect in RS-INs is sufficiently proven.

This point is related to Reviewer 1 points 2, 3 and 4. In the experiments with RS cell of Figure 4g (previous 4f), the number of cells is 10 wt vs 7 tg, which are of the same order of magnitude as in experiments with FS cells of Figure 1g (13 wt and 8 tg) for which the difference was significant. We performed a power analysis of the data of Figure 4g and determined that the sample size of 48 cells would be necessary to reach significance, which is biologically unrealistic and thus support our conclusion of no differences in RS cells. Results of the power analysis were added in Methods (line 295-296), Figure 4g legend, and the statistical table 1-1.

2. It is not clear whether the statistical analysis (Mann-Whitney test) used for data shown in Figs. 2f,m, 3 and 5d is appropriate (cf. Fig. 7e).

This point is related to Reviewer 1 point 1. As suggested by the reviewer 1, we revised our statistical analyses of the data for multiple comparisons of repeated measures. we tested for data normality and homoscedasticity (Shapiro-Wilk and Levene tests respectively). If data data passed normality and homoscedasticity assumptions, we used a mixed repeated-measure ANOVA to test for significant difference between genotypes (ex. Figure 1e). We did not perform multiple Bonferroni pair-wise comparisons, but measured the linear regression of repeated measures to obtain the gain (slope) of input-output functions (cell firing or synaptic current) and compared between genotypes (ex. Figure 1e and f). If normalcy test failed, we used one-way Friedmann ANOVA to determine significance of repeated-measures for each genotype (time effect; ex Figure 3a) and followed by a Mann-Whitney test for a single pair-wise comparisons of the last data pair (end of time series) to test for significant difference between genotypes (ex. Figure 3a). The text was modified accordingly in the Methods (lines 212-214, 292), in the statistical table (Table1-1), and in Figure legends 1-7.

3. The authors used PV-cre/Syngap1flox mice for the comparison of cell loss. It would be interesting to check FS-IN properties and excitatory responses in pyramidal cells in those mice.

We agree with the reviewer that comparing the neural properties of PV-cre/Syngap1flox mice would be interesting, but we considered these additional experiments beyond the scope of the present paper.

4. The authors should define meaningful abbreviations for the mouse strains used and use them consistently throughout the manuscript. Using “Tg(Nkx2.1-Cre);RCEflox/flox;Syngap1flox/+ and Tg(Nkx2.1-Cre);RCEflox/flox;Syngap1+/+” is not reader-friendly and switching between “CTRL”, “WT”, cHET” and other nomenclature is misleading.

As suggested by the reviewer, we defined meaningful abbreviations as listed below for the mouse strains in Methods (lines 129-140) and used them throughout the manuscript: 8

- Tg(Nkx2.1-Cre);RCEflox/flox;Syngap1flox/+ (referred herein as Nkx2.1-Syngap1f/+)

- Tg(Nkx2.1 Cre);RCEflox/flox;Syngap1+/+ (referred herein as Nkx2.1-Syngap1+/+)

- Tg(PVCre);RCEflx/flx;Syngap1flox/+ (referred herein as Pv-Syngap1f/+)

- Tg(PVCre);RCEflx/flx;Syngap1+/+ (referred herein as Pv-Syngap1+/+)

- Tg(Emx1Cre);Syngap1flox/+ (referred herein as Emx1-Syngap1f/+)

- Tg(Emx1Cre);Syngap1flox/flox (referred herein as Emx1-Syngap1f/f)

- Tg(Emx1Cre);Syngap1+/+ (referred herein as Emx1-Syngap1+/+)

5. Explain numbers given in summary graphs (e.g., “13 (6)”).

As requested by the reviewer, we explained in each legend of the Figures that the number in the summary graphs correspond to numbers of cells and mice.

6. Figs. 3c,d: Is there direct statistical evidence for the rescue of short-term facilitation by BAPTA in cHET cells?

This point is related to Reviewer 1 point 5. As requested by the reviewer 1 (and as indicated in point 1 above with the revised statistical analysis), we clarified and phrased more carefully the conclusions of the results of short-term facilitation in Figures 3 and 6. We clarified the results testing for short-term plasticity in each genotype (using Friedman ANOVA), and testing for a difference between genotypes with a single pairwise comparison of EPSC amplitude ratio for stimulus #10 at the end of the train (using a Mann-Whitney test). Our results indicate that short-term facilitation in FS cells is impaired in Nkx2.1-Syngap1f/+ mice in Figure 3, and that short-term facilitation in RS cells is mostly unchanged in Nkx2.1-Syngap1f/+ mice in Figure 6.

We modified accordingly the text in the Results (lines 366-369; 427-430), Figures 3 and 6 and their respective legends, and the statistical table 1-1.

We clarified in the legend of Figure 3c and d, that the EPSC amplitude ratio for the stimulus number 10 in summary plots show significant difference between genotypes in 3c but not in 3d, indicating rescue of short-term facilitation by BAPTA in Nkx2.1+ FS-INs from Nkx2.1- Syngap1f/+ mice in 3d.

Note that for the BAPTA experiments in Fig. 3d, although the data was normally distributed, we used the non-parametric statistical analyses (Friedman ANOVAs and Mann-Whitney test) for comparing results with those of Fig. 3c (and indicating rescue by BAPTA). Using a parametric statistical analysis yielded the same results (significant facilitation and no difference between genotypes).

Minor:

Define the criteria for “stable” holding currents and series resistance. The criteria were defined in the Methods (line 180). l. 224: define temperature. Temperature was defined (now line 227, 232) l. 227: No wash steps between primary and secondary antibody incubation? Correction made (now line 235) l. 231: Provide correct information for the anti-chicken antibody used. Correction made (now 9 line 240) l. 240: Explain “PV-GFPhg”. Correction made (now line 250) l. 414: INs Correction made (now line 417) l. 1073: “c-f” Correction made (now line 1112) l. 1087: define Cre-promoter line Correction made (now line 1126) “EGFP” vs. “GFP” Correction made “flox” vs. “flx” Correction made
