# Supplementary material for: Syngap1 Disruption Induced by Recombination between Inverted loxP Sites Is Associated with Hippocampal Interneuron Dysfunction
Source: eNeuro. 2023 May 4;10(5):ENEURO.0475-22.2023. doi: 10.1523/ENEURO.0475-22.2023 (PMC10166128; doi:10.1523/ENEURO.0475-22.2023)
Supplement: Extended Data Figure 1-1 — Statistical summary table. Download Figure 1-1, DOC file. [file enu-eN-NWR-0475-22-s02.doc]

**Extended Data Figure 1-1. Statistical summary Table.**

| Figure | Data structure (distribution) | Type of test | *p* value |
| --- | --- | --- | --- |
| 1b | Normal | Unpaired t-test | 0.0063 |
| 1c | Normal | Unpaired t-test | 0.54 |
| 1e | Normal | Mixed repeated-measures ANOVA | F _(1, 19 genotype)_ = 7.272 *p*=0.0143  F (_1.677, 31.86_ _time_) = 120.7, *P<0.0001* |
| 1f | Normal | Unpaired t-test | 0.012 |
| 1g | Normal | Unpaired t-test | 0.02 |
| 2b | Not normal | Mann-Whitney test | 0.66 |
| 2c | Normal | Unpaired t-test | 0.001 |
| 2d | Not normal | Mann-Whitney test | 0.002 |
| 2f | Normal | Mixed repeated-measures ANOVA | F _(1, 31 genotype)_ =11.6 *p*=0.001  F (_1.307, 40.53 time_) = 97.28  *p<0.0001* |
| 2g | Normal | Unpaired t-test | 0.003 |
| 2i | Not normal | Mann-Whitney test | *<0.0001* |
| 2j | Not normal | Mann Whitney test | 0.002 |
| 2k | Normal | Unpaired t-test | 0.23 |
| 2m | Normal | Mixed repeated-measures ANOVA | F _(1, 17 genotype)_ =1.9 *p*=0.18  F (_1.150, 19.55 time_) =21.07  *p<0.0001* |
|  |  |  |  |
| 2n | Normal | Unpaired t-test | 0.0814 |
|  |  | Power analysis :  Sample size to reach significance | Overall n=54 |
| 3a | Not normal | Friedman ANOVA | WT *p*= 0.003  cKO *p*<0.0001 |
|  |  | Mann-Whitney  Normalized EPSC at stim 10 | 0.04 |
| 3b | Not normal | Friedman ANOVA | WT *p*= 0.0002  cKO *p*<0.0001 |
|  |  | Mann-Whitney  Normalized EPSC at stim 10 | 0.02 |
| 3c | Not normal | Friedman ANOVA | WT *p*<0.0001  cKO *p*<0.0001 |
|  |  | Mann-Whitney  Normalized EPSC at stim 10 | 0.01 |
| 3d | Normal | Friedman ANOVA | cKO no BAPTA *p*<0.0001  cKO BAPTA *p*=0.0005 |
|  |  | Mann-Whitney  Normalized EPSC at stim 10 | 0.49 |
| 4b | Not normal | Mann Whitney test | 0.06 |
| 4c | Not normal | Mann Whitney test | 0.61 |
| 4e | Normal | Mixed repeated-measures ANOVA | F (_1, 15 genotype_) = 0.7545 *p*=0.398  F (_1.571, 23.56 time_) = 82.31 *p*<0.0001 |
| 4f | Normal | Unpaired t-test | 0.64 |
| 4g | Normal | Unpaired t-test | 0.09 |
|  |  | Power analysis :  Sample size to reach significance | Overall n=48 |
| 5b | Normal | Unpaired t-test | 0.32 |
|  |  | Power analysis :  Sample size to reach significance | Overall n=106 |
| 5c | Normal | Unpaired t-test | 0.14 |
|  |  | Power analysis :  Sample size to reach significance | Overall n=118 |
| 5d | Normal | Mixed repeated-measures ANOVA | F (_1, 30 genotype_) = 3.78 *p*=0.06  F (_1.415, 42.45 time_) = 82.31 *p*<0.0001 |
| 5e | Normal | Unpaired t-test | 0.09 |
|  |  | Power analysis :  Sample size to reach significance | Overall n=104 |
| 6a | Not Normal | Friedman ANOVA | WT *p*= 0.002  cKO *p*=0.0002 |
|  |  | Mann-Whitney  Normalized EPSC at stim 10 | 0.15 |
| 6b | Not Normal | Friedman ANOVA | WT *p*= 0.0002  cKO *p*=0.0002 |
|  |  | Mann-Whitney  Normalized EPSC at stim 10 | 0.19 |
| 6c | Not Normal | Friedman ANOVA | 0.16 |
|  |  | Mann-Whitney  Normalized EPSC at stim 10 | 0.03 |
| 7b | Not normal | Mann-Whitney test | 0.02 |
| 7c | Not normal | Mann-Whitney test | 0.005 |
| 7e | Normal | Mixed repeated-measures ANOVA | F _(1, 14 genotype)_ = 6.105, *p*=0.026  F (_1.294, 18.11 time_) = 18.78, *p*=0.0002 |
| 8 b1 | Normal | Unpaired t test | 0.005 |
| 8b2 | Normal | Unpaired t test | 0.029 |
| 8d | Normal | Unpaired t test | 0.298 |
