# Supplementary figures and images for: Syngap1 Disruption Induced by Recombination between Inverted loxP Sites Is Associated with Hippocampal Interneuron Dysfunction
Source: eNeuro. 2023 May 4;10(5):ENEURO.0475-22.2023. doi: 10.1523/ENEURO.0475-22.2023 (PMC10166128; doi:10.1523/ENEURO.0475-22.2023)

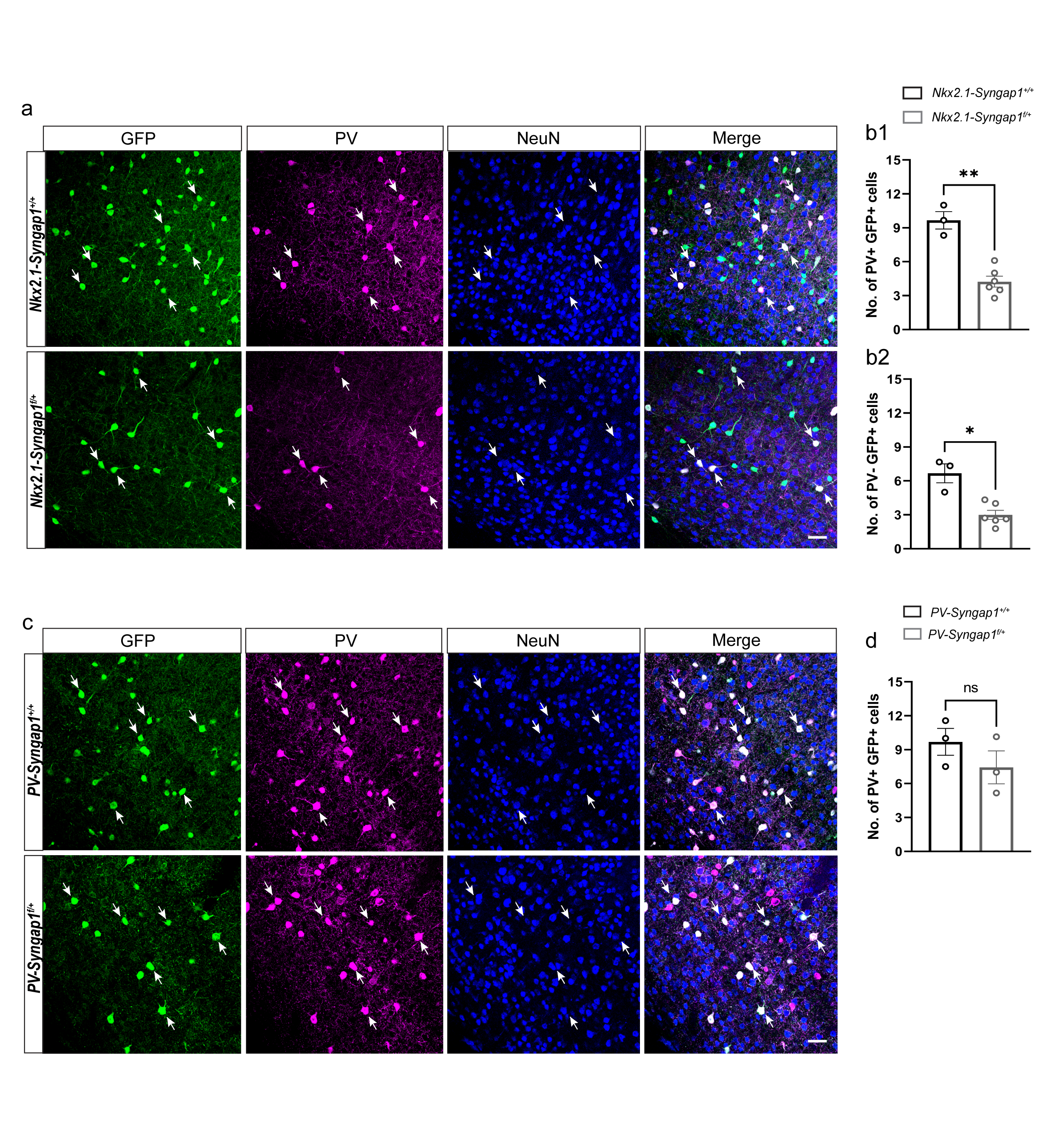

Supplement: Extended Data Figure 8-1 — Number of PV interneurons in the somatosensory cortex of PV-Syngap1f/+ and Nkx2.1-Syngap1f/+ mice. a, Confocal images of coronal sections from the somatosensory cortex (layer 5/6) of Nkx2.1-Syngap1+/+ and Nkx2.1-Syngap1f/+ of adult mice immunostained for GFP (green), PV (magenta), PV/GFP (white), and NeuN (blue). b1, Quantification of the number of PV+GFP+ cells in the somatosensory cortex (layer 5/6) of Nkx2.1-Syngap1+/+ (n = 3) and Nkx2.1-Syngap1f/+ (n = 6) adult mice. The number of PV+GFP+ were significantly reduced in Nkx2.1-Syngap1f/+ when compared to the Nkx2.1-Syngap1+/+ (unpaired t test with Welch’s correction, *p = 0.0052). b2, Quantification of the number of PV-GFP+ cells in the somatosensory cortex (layer 5/6) of Nkx2.1-Syngap1+/+ (n = 3) and Nkx2.1-Syngap1f/+ (n = 6) adult mice. The number of PV-GFP+ were significantly reduced in Nkx2.1-Syngap1f/+ when compared to the Nkx2.1-Syngap1+/+ mice (Unpaired t test with Welch’s correction, *p = 0.0299). c, Confocal images of coronal sections from the somatosensory cortex (layer 5/6) of PV-Syngap+/+ and PV-Syngap1f/+ of adult mice immunostained for GFP (green), PV (magenta), PV/GFP (white), and NeuN (blue). d, Quantification of the number in the somatosensory cortex (layer 5/6) of PV+ GFP+ cells in PV-Syngap1+/+ (n = 3) and PV-Syngap1f/+ (n = 3). The number of PV+ GFP+ was not significantly different in PV-Syngap1f/+ when compared to PV-Syngap1+/+ adult mice (unpaired t test with Welch’s correction, p = 0.2981). Download Figure 8-1, TIF file. [file enu-eN-NWR-0475-22-s03.tif]
